# Novel Approaches for Elongation of Fish Oils into Very-Long-Chain Polyunsaturated Fatty Acids and Their Enzymatic Interesterification into Glycerolipids

**DOI:** 10.1021/acs.jafc.3c05355

**Published:** 2023-11-10

**Authors:** Tereza Honzíková, Martin-Paul Agbaga, Robert Eugene Anderson, Richard Brush, Mohiuddin Ahmad, Lenka Musílková, Karolína Šejstalová, Katsiaryna Alishevich, Radek Beneš, Petra Šimicová, Markéta Berčíková, Vladimír Filip, Jan Kyselka

**Affiliations:** † Department of Dairy, Fat and Cosmetics, Faculty of Food and Biochemical Technology, University of Chemistry and Technology, Technická 3, 166 28 Prague, Czechia; ^‡^Departments of Cell Biology & ^§^Ophthalmology, Dean McGee Eye Institute, University of Oklahoma Health Sciences Center, Oklahoma City, Oklahoma 73104, United States; ∥ The Department of Chemistry of Natural Compounds, Faculty of Food and Biochemical Technology, University of Chemistry and Technology, Technická 5, 166 28 Prague, Czechia

**Keywords:** very-long-chain polyunsaturated fatty acids, fish oil concentrate, enzymatic interesterification, lipases, ELOVL4, eicosapentaenoic acid, docosahexaenoic acid, green chemistry

## Abstract

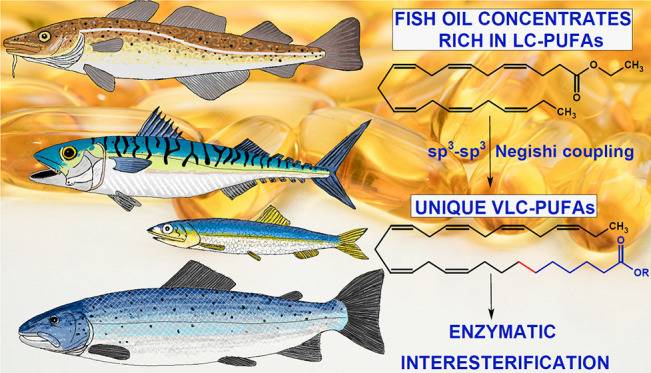

Elongation of the Very-Long-Chain Fatty Acids-4 (ELOVL4) enzyme that is expressed in neuronal tissues, sperm, and testes mediates biosynthesis of very-long-chain polyunsaturated fatty acids (VLC-PUFAs) from dietary long chain PUFAs (LC-PUFAs). The VLC-PUFAs are critical for neuronal and reproductive function. Therefore, mutations in ELOVL4 that affect VLC-PUFA biosynthesis contribute to retinal degenerative diseases including Autosomal Dominant Stargardt-like Macular Dystrophy (STGD3). Recent studies have also shown not only a depletion of retinal VLC-PUFAs with normal aging but also a more significant loss of VLC-PUFAs in donor eyes of patients with age-related macular degeneration (AMD). However, currently, there are no natural sources of VLC-PUFAs to be evaluated as dietary supplements for the attenuation of retinal degeneration in animal models of STGD3. Here, we report the development of a novel chemical approach for elongation of eicosapentaenoic (C20:5 *n*-3) and docosahexaenoic (C22:6 *n*-3) acids from fish oils by 6 carbon atoms to make a unique group of VLC-PUFAs, namely *all-cis*-hexacosa-11,14,17,20,23-pentaenoic acids (C26:5 *n*-3) and *all-cis*-octacosa-10,13,16,19,22,25-hexaenoic acids (C28:6 *n*-3). The three-step elongation approach that we report herein resulted in a good overall yield of up to 20.2%. This more sustainable approach also resulted in improved functional group compatibility and minimal impact on the geometrical integrity of the *all*-*cis* double bond system of the VLC-PUFAs. In addition, we also successfully used commercial deep-sea fish oil concentrate as an inexpensive material for the C6 elongation of fish oil LC-PUFAs into VLC-PUFAs, which resulted in the making of gram scales of VLC-PUFAs with an even higher isolation yield of 31.0%. The quality of fish oils and the content of oxidized lipids were key since both strongly affected the activity of the PEPPSI-IPr catalyst and ultimately the yield of coupling reactions. Downstream enzymatic interesterification was used for the first time to prepare structured glycerolipids enriched with VLC-PUFAs that could be evaluated *in vivo* to determine absorption and transport to target tissues relative to those of the free fatty acid forms. It turned out that in the synthesis of structured triacylglycerols and glycerophospholipids with VLC-PUFAs, the polarity of the immobilized lipase carrier and its humidity were essential.

## 1 Introduction

**Figure 1:**
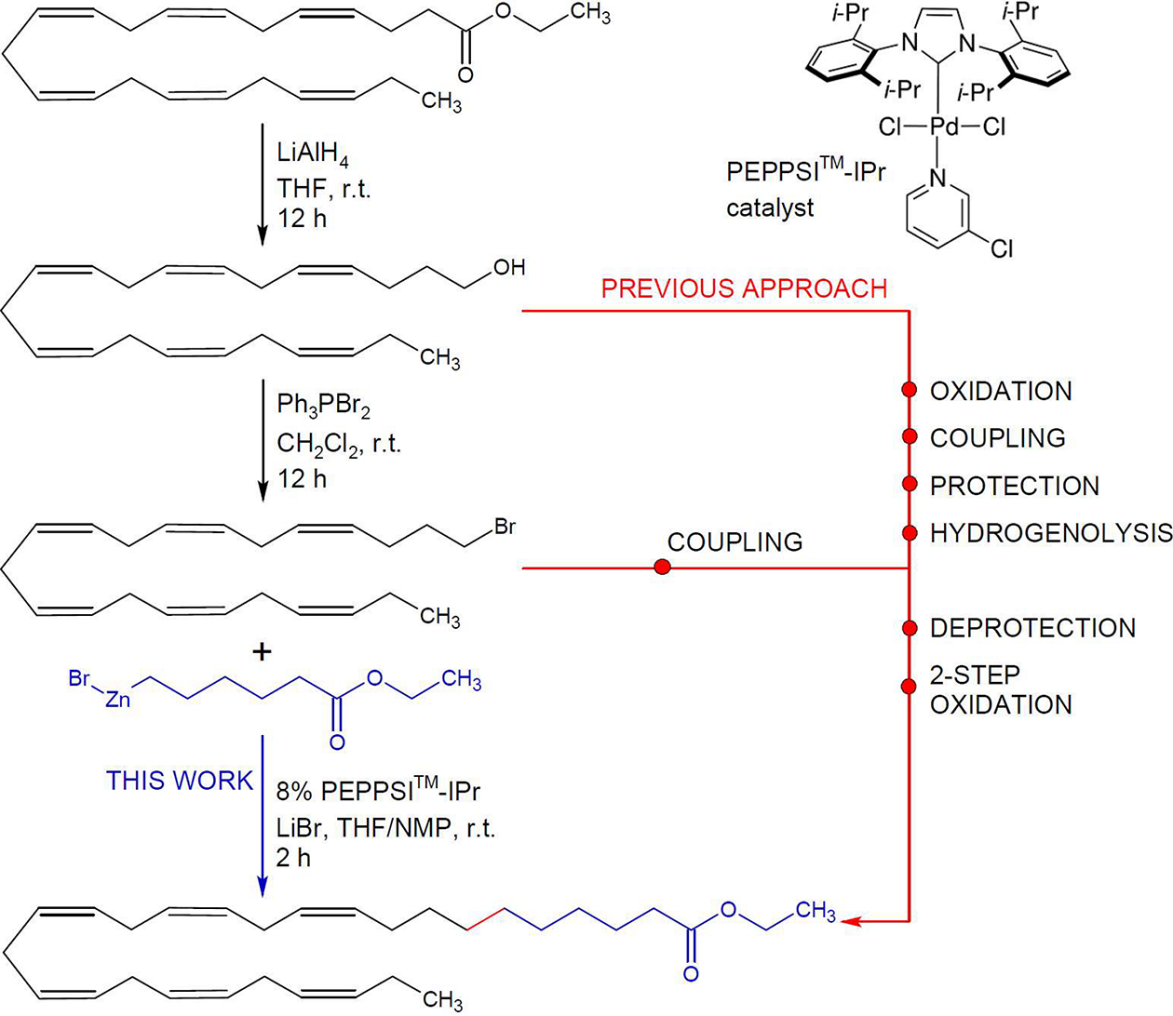
Conventional and newly proposed elongation of eicosapentaenoic (C20:5 *n*-3) and docosahexaenoic (C22:6 *n*-3) acids building blocks derived from fish oils.

Long-chain polyunsaturated fatty acids (LC-PUFAs; C20–C24) are physiologically important lipids that are essential for maintaining the cellular structure and function. These fatty acids are deemed “essential” because they cannot be synthesized by mammals and, thus, require a dietary source. There are two classes of essential PUFAs identified as *n*-3 and *n*-6; this designation is based on the position of the first double bond from the methyl terminal end of the fatty acid chain. Essentially all PUFAs found in natural sources have *cis* double bonds that are methylene interrupted (see Figure 1). Interestingly, mammals cannot interconvert polyunsaturated fatty acids (PUFAs) between the *n*-3 and *n*-6 series.^1^

In mammals, LC-PUFAs represented by arachidonic acid (AA, C20:4 *n*-6), eicosapentaenoic acid (EPA, C20:5 *n*-3), and docosahexaenoic acid (DHA, C22:6 *n*-3) play pivotal roles in a variety of cellular processes. For instance, LC-PUFAs of *n*-3 series are known to be anti-inflammatory, while endogenously produced eicosanoids derived from *n*-6 arachidonic acid promote the inflammatory response.^2,3^ Also, the delicate balance in the blood-clotting cascade can be influenced by the *n*-3 PUFA levels in the blood.^4^ It is worth noting that *n*-3 LC-PUFAs are also responsible for the normal development of rod function in the retina as described in the 1970s by the Anderson laboratory,^5,6^ which provided the first direct evidence of a physiological role for *n*-3 PUFAs in a cellular function. They discovered that dietary supplementation of *n*-3 PUFAs in rats enhanced the electroretinographic response (retinal function), while *n*-6 PUFAs had a slight effect and *n*-9 PUFAs were ineffective. It is now widely accepted that the supplementation of infant and prenatal formulas with DHA and AA improves the development of the children’s brain and retina.^7,8^

In the past few decades, lipid researchers focused primarily on the essential functions of *n*-6 and *n*-3 LC-PUFAs. Largely overlooked was another class of PUFAs called very-long-chain (VLC)-PUFAs, which contain *n*-3 and *n*-6 fatty acids from 26 to 40 carbons or more in length. In 1987, Aveldaño and Sprecher first reported the presence of VLC-PUFAs on the *sn*-1 position of phosphatidylcholine (PC) in bovine retinal photoreceptor membranes.^9−11^ In the same year, Poulos’ lab reported the presence of VLC-PUFAs in sphingomyelin of rat spermatozoa.^12^ However, the physiological relevance of VLC-PUFAs was not known until Agbaga et al.^13^ discovered that the mutation that causes Autosomal Dominant Stargardt-like Macular Dystrophy-3 (STGD3), a juvenile form of macular degeneration, was in the gene that encoded the enzyme responsible for the biosynthesis of VLC-PUFAs. Several laboratories had identified different mutations in a gene called Elongation of Very-Long-Chain Fatty Acids-4,^14−16^ but its function remained unknown. Agbaga et al.^13^ found that ELOVL4 also synthesizes VLC saturated fatty acids (VLC-SFAs) of 28–32 carbons in length. VLC-SFAs are found in brain,^17,18^ skin,^19^ and Meibomian glands.^20^

In mammals, while VLC-PUFAs occur naturally in ELOVL4-expressing tissues, they are rarely consumed in a normal vertebrate diet, yet they are uniquely present in the retina^9,10^ and gonads^12^ of vertebrates. Compared to the retina and sperm, although ELOVL4 is expressed throughout the brain,^21^ the Agbaga/Anderson lab searched exhaustively for VLC-PUFAs in the mouse brain and found none.^22^ However, they were able to identify very-long-chain saturated fatty acids, which are also synthesized by ELOVL4, in sphingolipids from synaptic vesicle preparations from the brain.^18^ In the retina and sperm, VLC-PUFAs are typically found as components of more complex lipid species such as glycerophospholipids, sphingolipids, and cerebrosides, which play a clinically significant role in the development of retinal function, nervous system, and reproduction processes in vertebrates. The distribution of VLC-PUFAs is tissue-specific, as is the expression of ELOVL4. ELOVL4 is not expressed in the liver,^23^ the site of DHA biosynthesis,^24^ and there are no VLC-PUFAs in the blood, indicating that the unique and limited distribution of VLC-PUFAs is due to *in situ* synthesis and that these fatty acids are not redistributed to other tissues. In general, enzymatic fatty acid elongation is a four-step process including (i) condensation, (ii) reduction, (iii) dehydration, and (iv) reduction, which provides the product with 2 carbon atoms added in each cycle.^25^ ELOVL4 catalyzes the rate-limiting Claisen condensation between acyl-CoA (≥26 carbon atoms) and malonyl-CoA regardless of their degree of unsaturation. The precursors of VLC-PUFAs, PUFAs C20–C22 with 4 to 6 double bonds, are obtained from the diet (e.g., fish oil) or biosynthesized in various tissues from essential α-linolenic acid (ALA; C18:3 *n*-3) and linoleic acid (LA; C18:2 *n*-6). Experiments in mice show that the biosynthesis of VLC-PUFA precursors is significantly predominant in the liver, as it contains the highest concentration and activity of the necessary enzymes (Δ^5^ and Δ^6^-desaturase, ELOVL2 and ELOVL5 elongase). Extrahepatic tissues show limited (brain, testes, and kidneys) or no (heart, lungs) activity of the relevant enzymes. At the same time, experiments show that although ALA and LA compete for the same enzymatic mechanism, there is a marked preference for the *n*-3 PUFA biosynthetic pathway, particularly for Δ^5^ and Δ^6^-desaturase and ELOVL2 elongase in liver, brain, testes, and kidney.^26^

VLC-PUFA-containing lipids are one of the least studied groups of lipids in the human body, yet their presence is essential for survival.^19^ This raises the question of why VLC-PUFAs are so exceptional. The biophysical properties of VLC-PUFAs may play a major role, which is related to their structure, being composed of a proximal part similar to a saturated fatty acid and a distal end with PUFA character (Figure 1). The extreme length (C_26–38_) and the degree of unsaturation can potentially lead to locally disordered glycerophospholipids in biomembranes, which could affect fluidity, permeability, fusion, molecular “flipping”, and membrane curvature. Thus, the loss of endogenously synthesized VLC-PUFAs could drastically alter the biophysical properties of biomembranes. VLC-PUFA deficiency is specifically associated with (i) Stargardt-like macular dystrophy (STGD3)^14−16^ and (ii) infertility.^27^ STGD3 is a disease with autosomal dominant inheritance caused by at least three different mutations in the ELOVL4 elongase-expressing gene.^14−16^ It manifests as a juvenile aggressive degeneration of the macular region of the retina, with rapid progression leading to early vision loss.

Since the LC-PUFAs 20:5 *n*-3, 22:5 *n*-3, and 22:6 *n*-3 can be elongated to VLC-PUFA,^13,28,29^ it seemed reasonable to suggest that providing these precursors in the diet of STGD3 patients may overcome the dominant negative effect of the mutated STDG3 gene product on VLC-PUFA production. Bernstein’s group at the Moran Eye Institute (UTAH) tested the hypothesis that dietary supplementation of 20:5 *n*-3 (650 mg/d) and 22:6 *n*-3 (350 mg/d) to a small number of their STGD3 patients could slow or prevent the development of STGD3 (ClinicalTrials.gov, #NCT00420602). However, their open-label clinical intervention trial did not attenuate the progression of retinal pathology, suggesting that these LC-PUFAs are not sufficient to prevent the progression of STGD3 in these patients.^30^ A similar conclusion was reached in the AREDS2 study involving a large cohort of patients with age-related macular degeneration (AMD).^31^ To circumvent the need for VLC-PUFA biosynthesis in the retina, the Bernstein group chemically synthesized 32:6 *n*-3 and administered it by gavage daily to a group of mice.^32^ They reported increased visual acuity and visual function (electroretinogram) in both wild-type mice and *Elovl4* rod-cone conditional knockout mice in comparison to controls. They concluded that the VLC-PUFAs bypassed the enzymatic elongation of fatty acids and enhanced retinal function.

The Bernstein studies raised the question of why VLC-PUFA supplements are not used commercially. There are at least three relevant reasons: (i) STGD3 is an orphan disease, (ii) no dietary sources exist, and (iii) current chemical synthetic approaches are rather difficult and expensive. Since STGD3 affects only a small number of individuals, there was little commercial interest in finding a source of VLC-PUFAs. In addition, recent findings by the Bernstein group relate retinal levels of VLC-PUFAs to AMD, which is a major cause of blindness in the aging world population. AMD risk is definitely associated with age, not with ELOVL4 genetic variants. Bernstein’s group reported that the levels of VLC-PUFAs were significantly reduced in the macula of age-matched donor eyes with AMD compared to nonaffected controls.^33^ They also showed that maculas from elderly non-AMD donors had significantly lower levels of VLC-PUFAs compared to middle-aged non-AMD donors.^33^ In support of Bernstein’s Lab findings, the Bazan lab recently showed that aging affects enzymatic pathways that elongate 22:6 *n*-3 to form VLC-PUFAs and that diminished VLC-PUFAs contribute to photoreceptor degeneration.^34^ Thus, the similarities between STGD3 and AMD-related changes in the reduction of macular VLC-PUFA levels, and possibly compromised supply of neuroprotective lipids derived from VLC-PUFAs (so-called elovanoids),^35^ greatly expand the patient base and make a strong case for finding a source of VLC-PUFAs that can be relatively inexpensive to make and would be available in sufficient quantities for long-term clinical trials for treating both STGD3 and AMD.

Numerous studies have shown the essential requirement for the *n*-3 and *n*-6 families of C-20 and C-22 PUFAs in a large number of fundamental biological processes.^35−39^ However, similar investigations of VLC-PUFA requirements have not been possible because these fatty acids are not available either in nature or via chemical synthesis. The need to treat STGD3 and the increasingly prevalent AMD with VLC-PUFAs is compelling and has the potential to drive market forces forward. To meet that need, we decided to contribute to the current state of knowledge by developing a sustainable, cost-effective, and simple organic synthesis of VLC-PUFAs using inexpensive building blocks, such as fish oil concentrates. Next, we aimed to provide new insights into enzymatic interesterification of prepared VLC-PUFAs with various triacylglycerols (TAG) and glycerophospholipids (PL).

## 2 Materials and Methods

### 2.1 Reagents and Materials

*all*-*cis*-Docosa-4,7,10,13,16,19-hexaenoic acid ethyl ester (DHA FAEE), *all*-*cis*-eicosa-5,8,11,14,17-pentaenoic acid ethyl ester (EPA FAEE), lithium aluminum hydride, triphenylphosphine dibromide, anhydrous tetrahydrofuran (THF), [1,3-bis(2,6-diisopropylphenyl)imidazol-2-ylidene](3-chloropyridyl)palladium(II) dichloride (PEPPSI-IPr catalyst), 6-ethoxy-6-oxohexylzinc bromide solution (0.5 M in THF), anhydrous 1-methyl-2-pyrrolidinone (NMP), lithium bromide ≥ 99%, ethylenediaminetetraacetic acid trisodium salt hydrate (Na_3_EDTA), 2-amino-2-methyl-1-propanol, 3-pyridinemethanol, trifluoroacetic anhydride, sodium methoxide, potassium *tert*-butoxide, and hexacosanoic acid were purchased from Sigma-Aldrich. Deep sea fish oil concentrate (FOC), Omega-3 Fish Oil Forte, was produced by MedPharma, Ltd. (Tetc̆ice, Czech Republic). A commercial sample of medium-chain triacylglycerols (MCT; fatty acid composition: octanoic acid 72% and decanoic acid 28%) was purchased from Aarhus Karlshamn Czech Republic, Ltd. (Prague). Synthetic standards of 1,2-dilauroyl-3-myristoyl-*rac*-glycerol (12–12–14) and ethyl hexacosanoate with 99% purities were prepared in our laboratory. All other solvents were reagent grade.

### 2.2 Preparation of Novel Ethyl *all*-*cis*-Hexacosa-11,14,17,20,23-pentaenoate (C26:5 *n*-3) and Ethyl *all*-*cis*-Octacosa-10,13,16,19,22,25-hexaenoate (C28:6 *n*-3) Standards

The approach selected in our laboratory was based on an innovative 3-step chemical elongation of commercially available ethyl esters of EPA and DHA. The current protocol included the reduction of LC-PUFA esters, conversion of primary alcohols to alkyl bromides, and the coupling of the alkyl bromides with short chain derivatives of saturated fatty acids via the Negishi reaction, which produced VLC-PUFAs (Figure 1). In brief, to a stirred solution of DHA FAEE (3.0 g, 8.41 mmol) in anhydrous THF (9.0 mL) under argon atmosphere was added dropwise a suspension of LiAlH_4_ (1.0 g, 26.22 mmol) in 18 mL of THF. After the mixture had been stirred for 12 h at 25 °C under inert conditions, ice-cold water was added to decompose an excess of LiAlH_4_. Next, the reaction mixture was taken up in Et_2_O (150 mL) and washed with a 5% HCl solution (2 × 100 mL) and distilled water (3 × 100 mL). The solvent was evaporated under reduced pressure, and the residue was worked up by silica gel chromatography using hexanes/Et_2_O (from 95:5 to 80:20, v/v) as eluent. *all*-*cis*-Docosa-4,7,10,13,16,19-hexaen-1-ol (DHA–OH) was obtained in 86.3% yield (2.3 g, 7.31 mmol). The structural analysis of DHA–OH (**1a**) is provided in Figure S1.

Bromination of DHA–OH (2.3 g, 7.31 mmol) was done under an argon atmosphere in a Schlenk reaction tube using (Ph)_3_PBr_2_ (3.9 g, 9.22 mmol) and 35 mL of dichloromethane. The bromination was carried out for 12 h at room temperature. Next, the mixture was taken up in dichloromethane (20 mL) and washed with distilled water (3 × 45 mL). After careful evaporation to dryness under reduced pressure, the residue was worked up by silica gel chromatography using hexane (450 mL) as eluent. *all*-*cis*-Docosa-4,7,10,13,16,19-hexaenyl bromide (DHA-Br) was obtained in 91.5% yield (2.5 g, 6.62 mmol). The structural analysis of DHA-Br (**2a**) is provided in Figure S2.

The final sp^3^-sp^3^ Negishi cross-coupling reaction was based on the protocol developed by Organ and co-workers.^40,41^ In brief, a three-neck round-bottom flask with a capacity of 100 mL was charged with the PEPPSI-IPr catalyst (362 mg, 0.53 mmol, 8 mol %), a solution of LiBr (4.4 mL, 3.02 M in THF, 13.29 mmol, 2 equiv), and NMP (2.2 mL). After the suspension was stirred for 10 min, the solution of 6-ethoxy-6-oxohexylzinc bromide (21.3 mL, 0.5 M in THF, 10.63 mmol, 1.6 equiv) was added, followed by dropwise addition of DHA-Br (2.5 g, 6.62 mmol, 1.0 equiv) in 10.6 mL of NMP. The reaction mixture was stirred at room temperature for another 2 h under argon atmosphere. After this time, the organic phase was taken up with 160 mL of Et_2_O and successively washed with 1.0 M Na_3_EDTA (100 mL), water (100 mL), and brine (100 mL). The solvent was evaporated under reduced pressure, and the mixture was worked up by column chromatography on silica gel (70–230 mesh) column to afford 749 mg of ethyl *all*-*cis*-octacosa-10,13,16,19,22,25-hexaenoate (C28:6 *n*-3) (eluted with hexane/ethyl acetate, from 100:0 to 98:2, v/v). Preparation of ethyl *all*-*cis*-hexacosa-11,14,17,20,23-pentaenoate (C26:5 *n*-3) starting from 1.0 g of EPA FAEE (3.03 mmol) was done in the same manner. All synthesized VLC-PUFAs were stored away from light under an argon atmosphere at −80 °C until structural analyses were carried out. Nuclear magnetic resonance (NMR) spectra (^1^H and ^13^C NMR APT) of compounds dissolved in deuterochloroform (CDCl_3_) were recorded on Bruker Avance 600 and Bruker Avance 500 (Bruker, Inc., Billerica, MA, USA). High resolution atmospheric-pressure chemical ionization (APCI-MS) mass spectra were recorded with an LC-MS LTQ-Orbitrap Velos (ThermoScientific, Waltham, MA, USA).

#### Ethyl *all-cis*-Octacosa-10,13,16,19,22,25-hexaenoate (**3a**)

Ethyl *all-cis*-octacosa-10,13,16,19,22,25-hexaenoate (DHA-C_6_H_12_–FAEE) was obtained in 20.2% (1.70 mmol, 749 mg) yield as a transparent, viscous liquid. R_f_ = 0.46 (hexane:Et_2_O:formic acid, 80:20:1, v/v/v). EI-MS, *m*/*z* (rel. int.) 440 [M]^+^ (1), 395 [M – OCH_2_CH_3_]^+^ (3), 371 [M −CH_2_–CH = CH–CH_2_–CH_3_]^+^ (4), 264 [M – (C_13_H_19_ + H)]^+^ (6), 108 [C_8_H_12_]^+^ (27); ^1^H NMR (500 MHz, CDCl_3_): δ 0.87–0.91 (t, 3H, C_j_-H_3_), 0.95–0.98 (t, 3H, C_a_-H_3_), 1.26–1.34 (m, 10H, C_e_-H_2_), 1.59–1.63 (p, 2H, C_d_-H_2_), 2.04–2.08 (p, 2H, C_i_-H_2_), 2.26–2.30 (m, 4H, C_f_-H_2_, C_c_-H_2_), 2.79–2.84 (t, 10H, C_h_-H_2_), 4.10–4.14 (k, 2H, C_b_-H_2_), 5.32–5.39 (m, 12H, C_g_–H). ^13^C NMR (126 MHz, CDCl_3_): δ 13.9 (C_a_), 14.25 (C_j_), 20.55 (C_i_), 25.54–25.63 (C_h_), 27.24 (C_f_), 29.14–29.62 (C_e_), 34.36–34.38 (C_c_), 60.12 (C_b_), 127.01–132.03 (C_g_), 173.86–173.89 (C_k_). APCI-MS *m*/*z* 441.37280 [M + H]^+^ (calcd for C_30_H_49_O_2_^+^ = 441.37271).

#### Ethyl *all*-*cis*-Hexacosa-11,14,17,20,23-pentaenoate (**3b**)

Ethyl *all-cis*-hexacosa-11,14,17,20,23-pentaenoate (EPA-C_6_H_12_–FAEE) was obtained in a 15.2% (0.46 mmol, 191 mg) yield as a transparent viscous liquid. R_f_ = 0.48 (hexane:Et_2_O:formic acid, 80:20:1, v/v/v). EI-MS, *m*/*z* (rel. int.) 414 [M]^+^ (1), 369 [M – OCH_2_CH_3_]^+^ (3), 345 [M – CH_2_–CH=CH–CH_2_–CH_3_]^+^ (5), 278 [M – (C_10_H_15_ + H)]^+^ (5), 108 [C_8_H_12_]^+^ (38); ^1^H NMR (500 MHz, CDCl_3_): δ 0.91–0.95 (t, 3H, C_j_-H_3_), 0.97–1.03 (t, 3H, C_a_-H_3_), 1.31–1.39 (m, 12H, C_e_-H_2_), 1.63–1.68 (p, 2H, C_d_-H_2_), 2.07–2.13 (p, 2H, C_i_-H_2_), 2.31–2.34 (m, 4H, C_f_-H_2_, C_c_-H_2_), 2.84–2.90 (dt, 8H, C_h_-H_2_), 4.14–4.18 (k, 2H, C_b_-H_2_), 5.35–5.46 (m, 10H, C_g_–H). ^13^C NMR (126 MHz, CDCl_3_): δ 13.9 (C_a_), 14.26 (C_j_), 20.55 (C_i_), 25.54–25.64 (C_h_), 27.26 (C_f_), 29.15–29.64 (C_e_), 34.36–34.40 (C_c_), 60.13 (C_b_), 127.02–132.03 (C_g_), 173.89 (C_k_). APCI-MS *m*/*z* 415.35715 [M + H]^+^ (calcd for C_28_H_47_O_2_^+^ = 415.35706).

### 2.3 Preparation of 3-Pyridylcarbinol Esters and 4,4-Dimethyloxazoline Derivatives of Major and Minor VLC-PUFA Isomers and Their GC-MS Analysis

Preparation of 3-pyridylcarbinol esters of VLC-PUFAs was done according to Destaillats and Angers.^42^ In brief, a solution of interesterification catalyst with a derivatization reagent was prepared by mixing 100 μL of a potassium *tert*-butoxide solution (1.0 M) with 200 μL of 3-pyridine methanol. The sample of VLC-PUFA ethyl esters (10 mg) was dissolved in anhydrous dichloromethane (1 mL) and combined with a fresh derivatization reagent in a closed vial. The reaction mixture was interesterified at 40 °C for 30 min. Next, it was taken up in hexanes (4 mL), washed with water (2 mL), and dried over anhydrous sodium sulfate. After evaporation to dryness under reduced pressure, the sample was dissolved in 1 mL of hexane for GC-MS analysis.

Mild preparation of 4,4-dimethyloxazolines (DMOX) of VLC-PUFA isomers for GC-MS analysis was done according to Svetashev.^43^ In brief, to a sample of VLC-PUFA esters (3 mg) were added a 50% solution of 2-amino-2-methyl-1-propanol in benzene (60 μL) and a fresh solution of 1% NaOCH_3_ in methanol (10 μL). Aminolysis of VLC-PUFAs was performed in a closed vial flushed with argon at 25 °C for 12 h in the dark. Next, acyl-2-methylpropanol amides were extracted by repeated partition between 0.5 mL of distilled water and 0.5 mL of hexane/Et_2_O (9:1, v/v). After evaporation to dryness under reduced pressure, the amides of VLC-PUFAs were converted into DMOX derivatives by incubation with 150 μL of trifluoracetic anhydride at 50 °C for 45 min. Afterward, evaporated samples were redissolved in hexanes (1.0 mL), washed with water (1.0 mL) to remove residual reagents, and dried over anhydrous sodium sulfate prior to GC-MS analysis.

Analysis of target compounds was performed on an Agilent 7820A gas chromatograph (Agilent Technologies, USA) coupled with a Mass Spectrometer (Agilent Technologies 5975 C VLMSD) and a 30 m HP-5MS capillary column (Agilent Technologies) 0.32 mm × 30 m, film thickness 0.25 μm. The conditions of analysis were as follows: hexane solution (≈1%) was used for the injection (1 μL), split injection (1:25) at 300 °C; the flow of carrier gas (He) was 1.0 mL/min; the oven temperature was programmed as follows: 80 °C (1/min); 80–320 °C (15 °C/min); 320 °C (20 C/min). EI mass spectra were recorded under an ionization voltage of 70 eV at 250 °C, and spectra were compared with the NIST library.

### 2.4 Scale-Up of VLC-PUFA Synthesis Starting from Fish Oil Concentrates

The commercial sample of deep-sea fish oil concentrate (FOC; 99.7 g, 108 mmol) was subjected to methanolysis by mixing with anhydrous methanol and potassium hydroxide in a molar ratio of 1:6:0.2. The reaction mixture was purged with argon and vigorously stirred at 25 °C for 3 h. The resulting fish oil fatty acid methyl esters (FOC-FAMEs) were separated from glycerol in a 1 L separatory funnel and washed thoroughly with a hot saturated NaCl solution to reach neutral pH. After drying over anhydrous sodium sulfate, molecular distillation was performed on a laboratory film evaporator FilmDist SP200 HT (Pilodist GmbH) at a temperature of 130 °C and a pressure of 0.0013 Pa; the distillation residue and the FOC-FAME distillate were cooled to 60 and 30 °C, respectively. A distillate aliquot (10.0 g) was worked up by silica gel chromatography using hexane to produce pure FOC-FAME (7.3 g, 22.09 mmol) free of oxidized lipids. The overall process is schematically shown in Figure S3. Purified samples of both FOC-FAME and commercial FOC were characterized by the determination of their fatty acid composition as well as key indicators of hydrolytic (acid value) and oxidative stability (peroxide value, iodine value, induction period) as previously described.^44^

Elongation of polyunsaturated esters was based on a previously developed protocol.^40,41^ Reduction of an aliquot of FOC-FAME (7.3 g, 22.09 mmol) by LiAlH_4_ provided fish oil fatty acid alcohols (FOC–OHs) in 89% yield (6.0 g, 19.73 mmol). Next, FOC–OH was subjected to bromination with Ph_3_PBr_2_ that yielded 93% of the product (6.7 g, 18.44 mmol) after purification by column chromatography. The last Negishi reaction was performed with a 1 g aliquot of fish oil alkyl bromides (FOC-Br) (2.75 mmol). Ethyl esters of the fish oil fatty acids elongated by 6 carbon atoms (FOC-VLC-PUFAs) were obtained in 31% yield (358 mg, 0.84 mmol). All synthesized FOC-VLC-PUFAs were stored under an argon atmosphere at −80 °C until structural characterization.

### 2.5 Enzymatic Interesterification of VLC-PUFAs with Triacylglycerols and Glycerophospholipids

Enzymatic interesterification of ethyl *all-cis*-octacosa-10,13,16,19,22,25-hexaenoate (C28:6 *n*-3) with commercial medium-chain triacylglycerols was done according to Haraldsson et al.^45^ Reaction conditions were modified according to Noel and Combes.^46^ In brief, the samples of MCT (7 mg, 14 μmol) and ethyl *all-cis*-octacosa-10,13,16,19,22,25-hexaenoate (21 mg, 48 μmol) were mixed in a 1:3 weight ratio in a 15 mL vial with a tightly fitting cap followed by the addition of 3 mL of hexane. After heating to 40 °C under an argon atmosphere, lipases with various selectivities (Table 1) were loaded at 10% by weight of the total lipids. Interesterifications were carried out for 40 h in the inert atmosphere of argon at 40 °C. After this time, the reaction mixtures were microfiltered, and the solvent was evaporated by a stream of argon. Internal standards, ethyl hexacosanoate (99%), and triacylglycerol 14–12–12 (99%) were added to quantify VLC-PUFA-enriched triacylglycerols. All samples were stored under argon atmosphere at −80 °C until structural analyses. Each enzyme was subjected to gravimetrical determination of water content using a Mettler Toledo HR 73 moisture analyzer. FTIR spectra (spectral region 4000–400 cm^–1^, 64 scans, resolution 4 cm^–1^) of the enzyme’s immobilization carrier were recorded on a Nicolet 6700 FTIR spectrometer (Thermo Scientific, Waltham, MA) using Omnic 8.0 software.

**Table 1:** Lipases Used for Enzymatic Interesterification of MCT Fat with VLC-PUFAs

**Lipase enzyme product name**	**Origin**	**Enzymatic activity**	**Immobilization carrier**	**Moisture content**^d^**[%]**	**Specifics**	**Producer State**
Lipozyme RM IM	*Rhizomucor miehei*	275 IUN^a^/g	Macroporous ion-exchange resin	0.70 ± 0.00	*sn*-1; *sn*-3	Novozymes Denmark
Lipozyme TL IM	*Thermomyces lanuginosus*	250 IUN^a^/g	Granulated silica gel	4.69 ± 0.10	*sn*-1; *sn*-3 (preferentially, but not uniquely)	Novozymes Denmark
Novozyme 435	*Candidaantarctica* B	10 000 PLU^b^/g	Macroporous acrylic resin	1.45 ± 0.05	Nonspecific	Novozymes Denmark
Lipase A *Candida antarctica*	Recombinant from *Aspergillus oryzae*	≥500 U^c^/g (lot result: 1590 U^c^/g)	Immobead 150 (copolymer of methacrylate)	2.04 ± 0.15	*sn*-2	Sigma-Aldrich Netherlands
Lipase B *Candida antarctica*	Recombinant from *Aspergillus oryzae*	≥1800 U^c^/g (lot result: 3273 U^c^/g)	Immobead 150 (copolymer of methacrylate)	1.65 ± 0.15	*sn*-3	Sigma-Aldrich Netherlands

a IUN = Interesterification Unit; 1 IUN is equal to 1 PLU.

b PLU = Propyl Laurate Unit; 1 PLU is the amount of enzyme activity which generates 1 μmol of propyl laurate per minute under defined standard conditions.

c 1 U corresponds to the amount of enzyme which liberates 1 μmol butyric acid per minute at pH 7.5 and 40 °C (tributyrin, Cat. No. 91010, as substrate).

d Water content measured on the Mettler Toledo HR 73 moisture analyzer.

The preparation of VLC-PUFA-enriched glycerophospholipids by enzymatic interesterification was carried out according to D’Arrigo and Servi.^47^ The reaction conditions were based on the report of Li et al.^48^ In brief, the samples of 1,2-dipalmitoyl-*sn*-glycero-3-phosphocholine (6 mg, 8 μmol) and ethyl *all-cis*-hexacosa-11,14,17,20,23-pentaenoate (18 mg, 44 μmol) were mixed in a 1:3 weight ratio in a 15 mL vial with a tightly fitting cap followed by the addition of 3 mL of hexane and flushing with argon. After heating to 45 °C, the lipases from *Rhizomucor miehei* and *Thermomyces lanuginosus* (Table 1) were added at 15% by weight of the total lipids. The argon atmosphere was renewed, and the reactions were carried out for 48 h at 45 °C. After this time, the reaction mixtures were microfiltered, and the solvent was evaporated by a stream of argon. All samples were stored under argon atmosphere at −80 °C until structural analyses. High resolution electrospray ionization (ESI-MS) spectra were recorded with an LC-MS LTQ-Orbitrap Velos (ThermoScientific, Waltham, MA, USA). We also separated glycerophosphatidylcholines by solid-phase extraction (SPE) to determine the equilibrium composition of the fatty acyl moieties. In brief, interesterified products were dissolved in 2 mL of hexane:diethyl ether (7:3, v/v) and applied to LC–Si SPE tubes (Supelclean, 6 mL, 1 g). Interfering FAEEs were removed by 6 mL of hexane:diethyl ether (7:3, v/v). Afterward, SPE tubes were washed with 10 mL of methanol, and analysis of released FAME was performed on an Agilent 8890N Gas Chromatograph (Agilent Technologies, USA) coupled with a flame-ionization detector (FID).

## 3 Results and Discussion

### 3.1 Preparation of Novel Ethyl *all*-*cis*-Hexacosa-11,14,17,20,23-pentaenoate (C26:5 *n*-3) and Ethyl *all*-*cis*-Octacosa-10,13,16,19,22,25-hexaenoate (C28:6 *n*-3) from EPA, DHA, and Fish Oil Concentrates

Deficiency of VLC-PUFAs in the human retina is associated with STGD3 and AMD, each of which results in blindness and thus strongly affects the quality of life. More recently, Bernstein and co-workers bypassed the physiological demand for VLC-PUFA synthesis by administering 32:6 *n*-3 to mice by gavage.^32^ Although dietary intake of VLC-PUFAs may represent a potential therapy for macular degeneration, no suitable dietary source has been found so far nor has biotechnological production based on non-GMO or GMO organisms. It is therefore clear that the organic synthesis of pure VLC-PUFAs for oral administration could provide a potentially effective treatment for AMD and STGD3, especially if they could be produced economically on a large scale while maintaining their characteristic *all-cis* geometrical configuration.

To date, current synthetic approaches are rather difficult, provide low yields, and are expensive for potential commercialization.^32,49^ Therefore, we decided to develop a sustainable, cost-effective, and simple organic synthesis of VLC-PUFAs using inexpensive building blocks with a minimum of required steps. In our study, we focused on the preparation of ethyl *all*-*cis*-octacosa-10,13,16,19,22,25-hexaenoate (**3a**, C28:6 *n*-3) and ethyl *all*-*cis*-hexacosa-11,14,17,20,23-pentaenoate (**3b**, C26:5 *n*-3), precursors of the VLC-PUFA. In our retrosynthetic analysis of **3a** and **3b**, a Pd-catalyzed sp^3^-sp^3^ cross-coupling reaction was selected as a key step leading back to an alkyl bromide (**2a**, **2b**) as an electrophile and a 6-ethoxy-6-oxohexylzinc bromide, which represented a commercial organometallic nucleophile. The proposed synthesis of VLC-PUFAs started with the reduction of readily available ethyl esters of EPA and DHA using an efficient protocol, as shown in Figure 1.

First of all, selected esters were reduced with LiAlH_4_ in THF to the corresponding fatty alcohols and purified by column chromatography with an overall yield of 86.3%. Next, polyunsaturated primary alcohols (**1a**, **1b**) were converted to alkyl bromides (**2a**, **2b**) using Ph_3_PBr_2_ in a nearly quantitative yield (91.5%). The last Pd-catalyzed sp^3^ (R-Br)-sp^3^ (R′-ZnBr) Negishi reaction was accomplished with only 8 mol % of the commercial PEPPSI-IPr catalyst, which was recently developed by Organ and co-workers.^40,41^ The total synthesis of **3a** and **3b** was achieved at room temperature by a user-friendly protocol with only 3 steps (Figure 1) in 15–21% overall (isolation) yield. In fact, this is even one step less than is required for VLC-PUFA biosynthesis.

**Figure 2:**
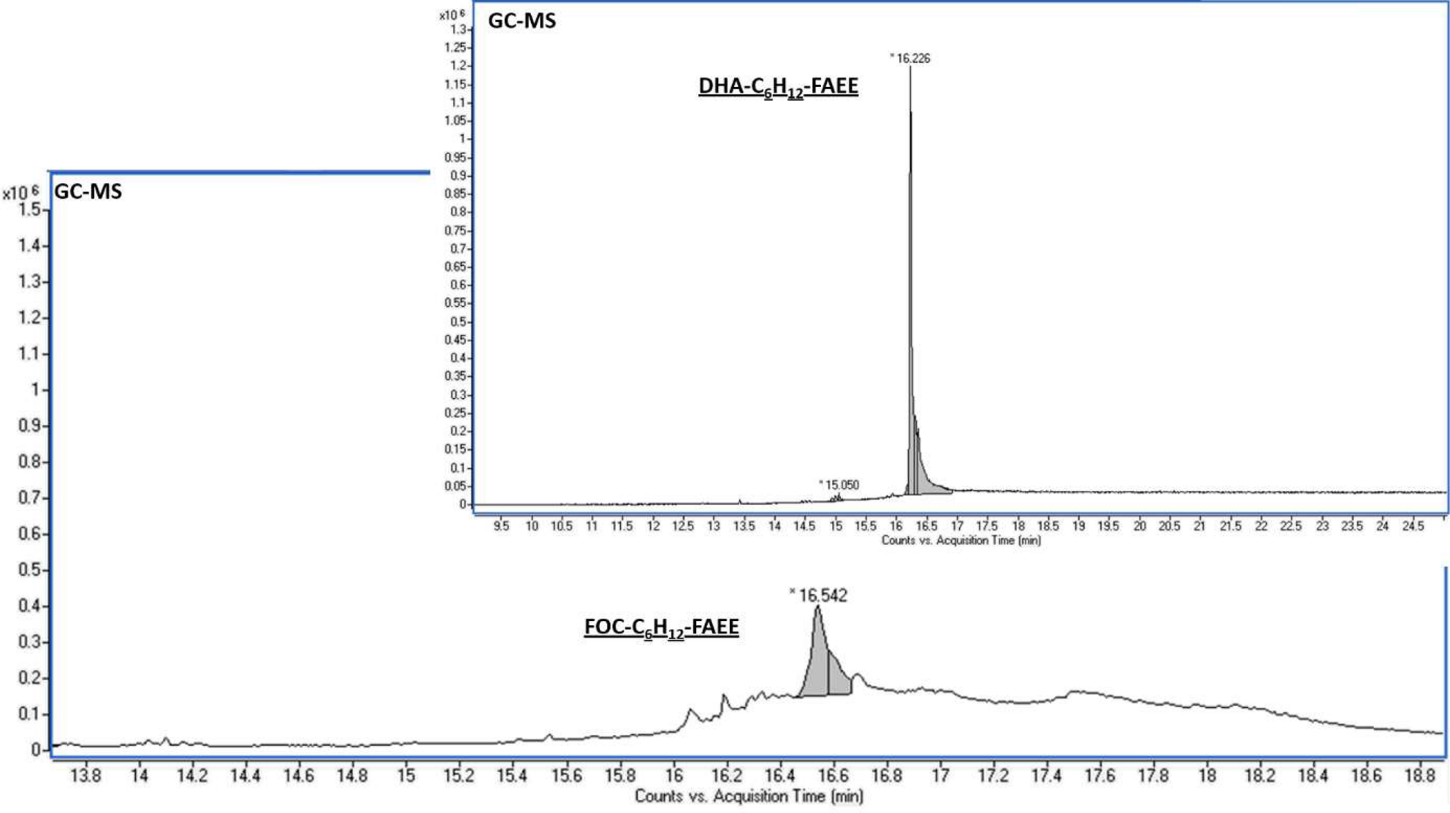
GC-based separation of major ethyl *all*-*cis*-octacosa-10,13,16,19,22,25-hexaenoate (C28:6 *n*-3) and its minor isomers prepared from DHA (upper image) and fish oil concentrates (lower image) on a 5% phenylmethyl polysiloxane column.

The major advantage of our simplified approach is improved functional group compatibility, increased isolation yield, and minimized impact of reagents on the geometric integrity of the *all*-*cis* double bond system of VLC-PUFAs (Figure 2). So far, previous protocols mostly employed Grignard reagents incompatible with esters, and thus, VLC-PUFAs required a protection, coupling, deprotection, and a two-step oxidation sequence (Figure 1, previous approach) and provided a lower yield (below 15%).^32,49^ Moreover, our selected approach can be readily modified to prepare the C32–36 VLC-PUFAs that are found in retinal membranes. To assess the sustainability of the developed synthetic approach reported herein, we used green metrics (Table 3) including the Atom Economy (AE) and the Process Atom Economy (PAE), which evaluate the environmental impact of our process. Next, we calculated the Environmental Factor (E-Factor), which allowed us to determine the quantity of all spent reagents, solvents, catalysts, and byproducts to synthesize 1 g of target ethyl *all*-*cis*-octacosa-10,13,16,19,22,25-hexaenoate (C28:6 *n*-3). Reaction schemes and calculations are shown in the Figures S4 and S5. As shown in Table 3, the three-step elongation reported herein allowed us a significant improvement of the AE (52.71%) and PAE (6.17%) compared to the synthesis of Goruspundi et al. (ref (32))/Wade et al. (ref (49)), indicating that more DHA used as the starting material was transformed into final product C28:6 *n*-3. In the case of our study, E-Factor was reduced 2.5-fold compared to the one of Goruspundi et al. (ref (32))/Wade et al. (ref (49)), creating a lower amount of waste as well as byproducts (Table 3).^50^

Once we developed a sustainable elongation protocol with readily available reagents, we tried to scale up the synthesis of VLC-PUFAs starting from commercial deep sea fish oil concentrates. Physicochemical composition of FOC was summarized in Table 2. Analyses proved the standard quality of marine feedstock with low acid value (0.35 mg KOH·g^–1^), acceptable peroxide value (3.03 mequiv act. O·kg^–1^), and the induction period of 0.16 h. First, the sample of FOC transformed to methyl esters was subjected to molecular distillation on the film evaporator FilmDist SP200 HT (Pilodist GmbH) to generate a mixture more concentrated in PUFAs (62.90 wt % of EPA, 26.47 wt % of DHA). Next, the FOC-FAME distillate was worked up by column chromatography to remove oxidized lipids as indicated by the decrease of peroxide value to 1.18 mequiv act. O·kg^–1^. Three-step elongation of FOC-FAME provided fish oil fatty acids (FOC-VLC-PUFAs) elongated by 6 carbon atoms in a 31% isolation yield (Figure 2). Therefore, the removal of the oxidized FOC-FAME to a third afforded a 2-fold increase in the yield of the coupling reaction as it protected the active part of the PEPPSI-IPr catalyst formed *in situ*, namely, N-heterocyclic carbene ligand-Pd(0). Repeating the above reaction sequence three times showed the effective use of FOC for the first time as an inexpensive material for the preparation of unique VLC-PUFAs on a gram scale. In other words, from 3.00 g of fish oil derived alkyl bromides, we have successfully prepared 1.07 g of VLC-PUFAs (63% of C26:5 *n*-3 and 26% of C28:6 *n*-3).

**Table 2:** Physicochemical Characterization of Deep-Sea Fish Oil Concentrate (FOC) and its Methyl Esters (FOC-FAMEs)

	FOC	FOC-FAME	Fatty acid content of FOC-FAME [wt %]^d^	
**Peroxide value**^a^ [meq act. O·kg^–1^]	3.03 ± 0.01	1.18 ± 0.00	**EPA**	62.90
**Acid value**^b^ [mg KOH·g^–1^]	0.35 ± 0.03	0.19 ± 0.00	**DHA**	26.47
**Induction period**^c^ [h]	0.16 ± 0.01	0.12 ± 0.00	**ΣSFA**	5.87
**Saponification value** [mg NaOH·g^–1^]	174.18	173.68	**ΣMUFA**	4.03
**Iodine value** [g I·100g^–1^]	336.95	33614	**ΣPUFA**	90.10

a Peroxide value determined according to EN ISO 3960.

b Acid value determined according to EN ISO 660.

c Induction period measured on the 743 Rancimat by Metrohm at 120 °C.

d Abbreviations: SFA = saturated fatty acid (palmitic, stearic, eicosanoic, docosanoic acid), MUFA = monounsaturated fatty acid (palmitoleic, oleic, eicosenoic, docosenoic acid), and PUFA = polyunsaturated fatty acid (linoleic, linolenic, eicosapentaenoic, docosahexaenoic acid).

**Table 3:** Comparison of Green Metrics Including Atom Economy, Process Atom Economy, and Environmental Factor between the Synthetic Approach of Goruspundi et al. (ref (32))/Wade et al. (ref (49)) and the One Proposed Herein

**Green metrics**	**Goruspundi et al.^32^**/**Wade et al.^49^**	**Honzíková et al.**
**Atom Economy** [%]	49.72	52.71
**Process Atom Economy** [%]	0.87	6.17
**Environmental Factor** [g/g]	4133.54	1620.14

### 3.2 Comprehensive Characterization of Major and Minor VLC-PUFA Isomers by EI-MS, APCI-MS, and NMR Spectroscopy

**Figure 3:**
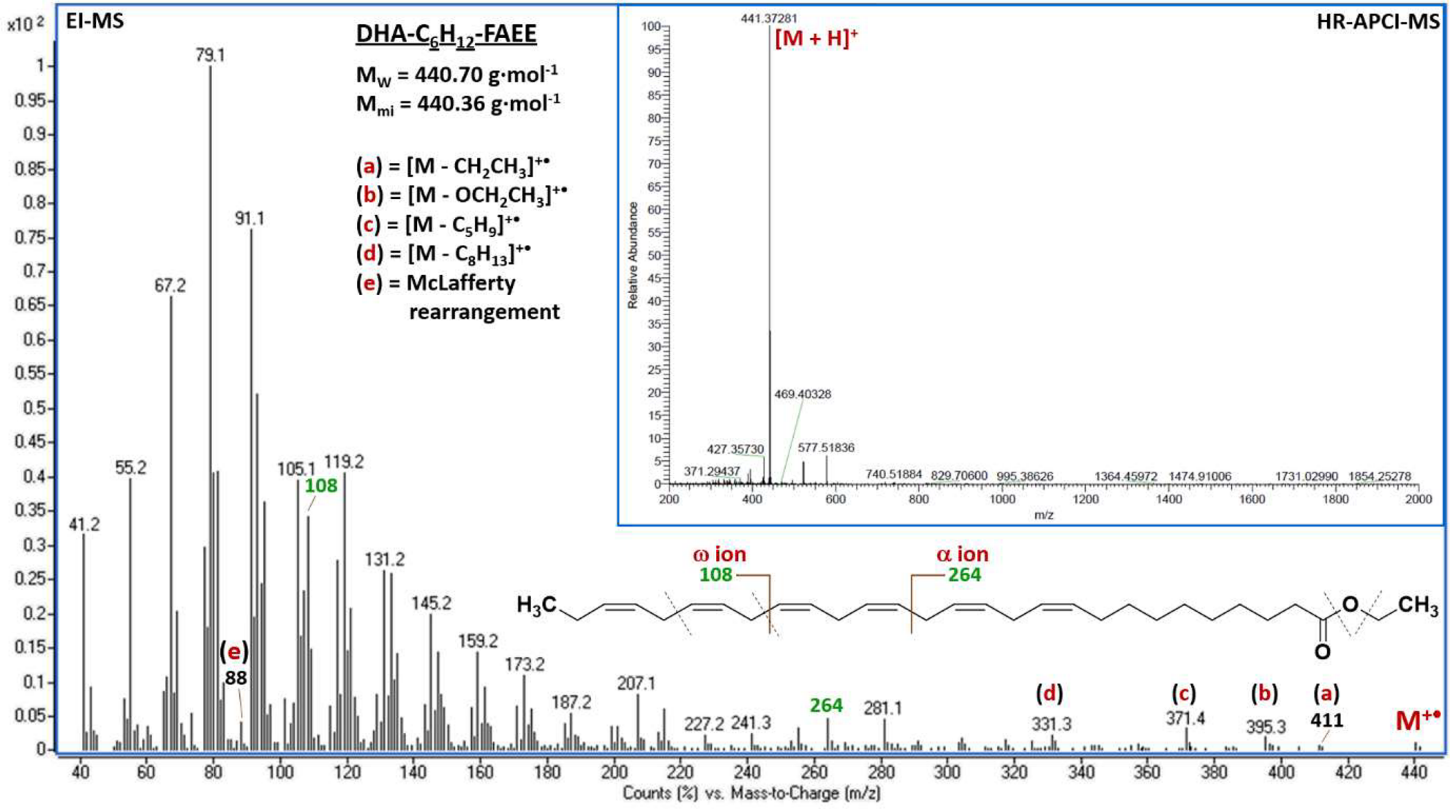
HR-APCI-MS (upper image) and EI-MS (lower image) analyses of ethyl *all-cis*-octacosa-10,13,16,19,22,25-hexaenoate (**3a**).

**Figure 4:**
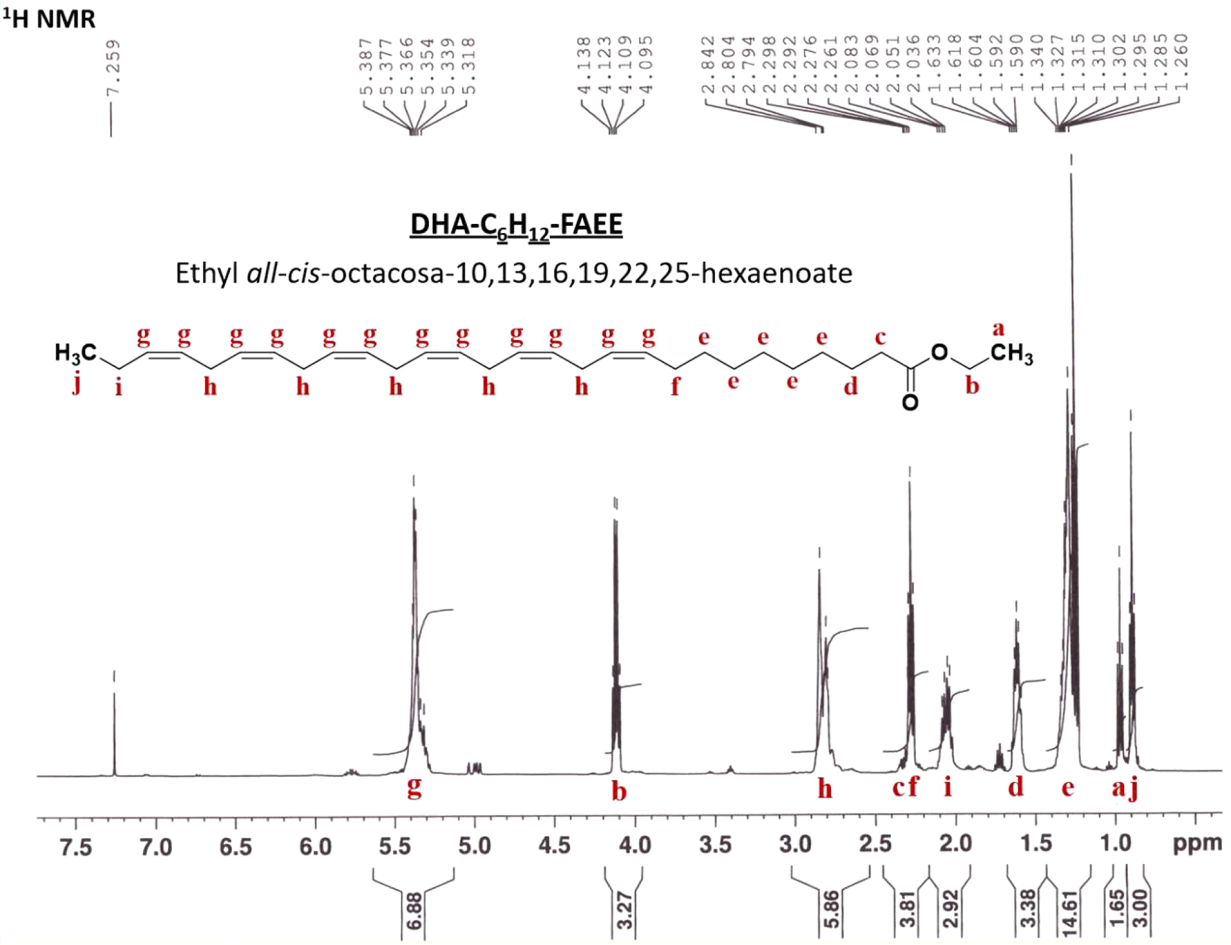
^1^H NMR analysis of ethyl *all-cis*-octacosa-10,13,16,19,22,25-hexaenoate (**3a**).

The structures of synthesized **3a** and **3b** were characterized by high-resolution APCI-MS, EI-MS, and NMR spectroscopy techniques. The results of GC-FID and NMR analysis confirmed at least 98% purity of both derivatives. Mass spectra obtained by APCI-MS allowed us to determine the accurate mass of protonated ethyl *all*-*cis*-hexacosa-11,14,17,20,23-pentaenoate (C26:5 *n*-3) and ethyl *all*-*cis*-octacosa-10,13,16,19,22,25-hexaenoate (C28:6 *n*-3) at *m*/*z* 415.35715 and 441.37281, respectively (upper image in Figure 3, Figure S6). In contrast to other mass spectrometry methods, APCI-MS was unable to distinguish between anti-inflammatory *n*-3 VLC-PUFAs and pro-inflammatory *n*-6 species associated with both decreased and increased AMD risk.^51^ For this purpose, synthesized standards of **3a** and **3b** were further analyzed by GC-MS. Although application of the EI source caused almost complete loss of molecular ions (M^+•^) at *m*/*z* 414.4 and 440.4, it also provided a highly diagnostic fragmentation pattern typical for *n*-3 VLC-PUFAs such as ω-ions and α-ions at *m*/*z* 108, 264, and 278 accompanied by α-cleavage (M^+•^ – OCH_2_CH_3_, M^+•^ – CH_2_CH_3_) and the McLafferty rearrangement (lower image in Figure 3, Figure S6). In addition, the diagnostic ion at *m*/*z* 151 typical for VLC-PUFAs of the *n*-6 series was completely absent. Next, elucidation of the VLC-PUFA *n*-3 structure was done by ^1^H NMR spectroscopy (Figure 4, Figure S7). The signal at δ 0.91 (t, 3H, 28-CH_3_) was assigned to H-28 protons at the terminal CH_3_ group. The chemical shifts for H-27 protons appeared at δ 2.04–2.08 (p, 2H, 27-CH_2_).

The combined results of GC-MS and GC-FID analyses obtained on elongated standards of both VLC-PUFAs and fish oil concentrates showed the presence of previously undescribed isomers of polyunsaturated VLC-FA with retention times longer than those of the corresponding *all*-*cis* derivatives (Figure 2). This raised the question whether these minorities (3–10%) represented positional or geometrical isomers. LC-MS coupled with ESI-MS and APCI-MS did not allow us to determine the localization of double bond positions along the VLC-PUFA carbon chain, while EI-MS of **3a** and **3b** in the form of ethyl esters suffered from extensive fragmentation and the absence of reference compounds in the mass spectral libraries. Therefore, we decided to prepare 3-pyridylcarbinol esters and 4,4-dimethyloxazolines of VLC-PUFAs to overcome the drawback of the identification of previously undescribed isomers. It is worth noting that mild reaction conditions with a temperature below 50 °C did not cause additional isomerization of our analytes.^42,43^

**Figure 5:**
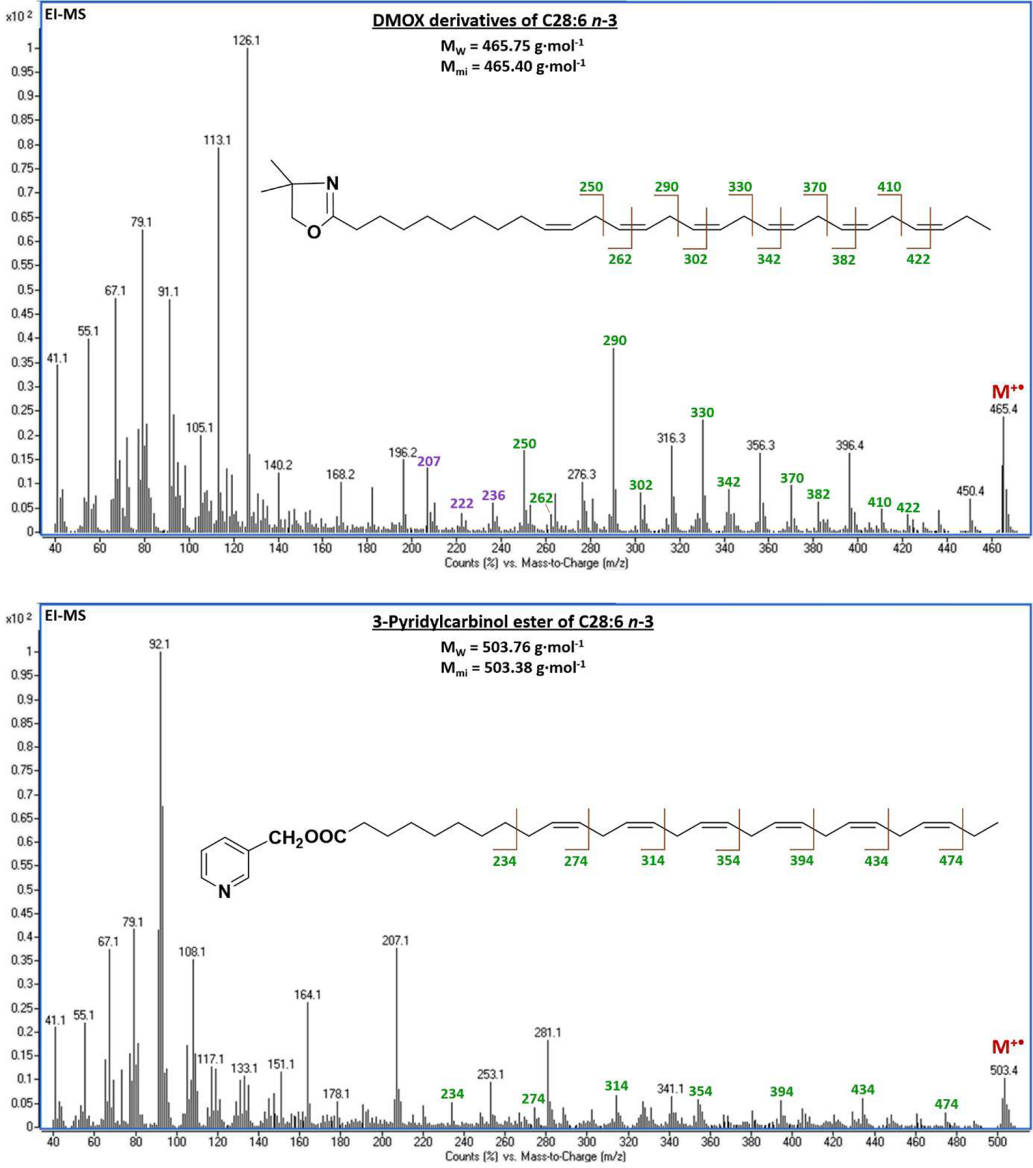
Mass spectra of minor isomers of ethyl octacosahexaenoate derived from DHA and fish oil concentrates in the form of 4,4-dimethyloxazoline (upper image) and 3-pyridylcarbinol ester (lower image) derivatives.

Representative isomers of C28:6 in the form of DMOX derivatives yielded a distinctive molecular ion at *m*/*z* 465.4 and an abundant ion at M^+•^ – 15 formed by the loss of the methyl radical either from the terminal position of VLC-PUFAs or the DMOX ring.^52^ All but the first double bond were recognized by the gaps of 12 amu between *m*/*z* = 250 and 262, 290 and 302, 330 and 342, 370 and 382, and 410 and 422, which readily located the monomethylene-interrupted double bond system in positions −13, −16, −19, −22, and −25, respectively (Figure 5, upper image). Distonic radical ions along with one hydrogen atom migration at *m*/*z* 126 (base), 140, 154, 168, 182, and 196 with a gap of 14 amu represented another group of diagnostic fragments formed by the cleavage of the saturated part of C28:6 isomers between positions 3 and 8. However, only the analysis of the 3-pyridylcarbinyl octacosahexaenoate provided unequivocal confirmation of the previously proposed structures. In the mass spectrum of 3-pyridylcarbinol ester of C28:6 (Figure 5, lower image), a gap of 40 amu at *m*/*z* = 234 to 274 to 314 to 354 to 394 to 434 clearly showed a fragmentation pattern of the double bond with the preceding CH_2_ group typical solely for geometrical isomers of 3-pyridylcarbinyl octacosa-10,13,16,19,22,25-hexaenoate. The achieved results were in good agreement with previously published data on LC-PUFAs.^42,43,52,53^ Mass spectra of the hexacosa-11,14,17,20,23-pentaenoate geometrical isomer in the form of 3-pyridylcarbinol esters and DMOX are provided in Figure S8.

To sum up, our synthetic approach using the PEPPSI-IPr organocatalyst only slightly affected the integrity of the *all*-*cis* double bond system. Structural analysis clearly refuted the conjugation of double bonds since we only confirmed the presence of VLC-PUFA geometrical isomers with a random distribution of *trans* double bonds.

### 3.3 New Insights into Nonspecific, 1,3-Regiospecific, and 2-Regiospecific Enzymatic Interesterification of VLC-PUFAs with Medium-Chain Triacylglycerols

VLC-PUFA ethyl esters made from fish oil concentrates and/or EPA/DHA, similar to adjuvant therapeutics Lovaza, can be used directly for oral administration.^54^ Particularly problematic is the low bioavailability of VLC fatty acids in the form of ethyl esters compared to triacylglycerols and glycerophospholipids. In natural glycerolipids, the stereospecific distribution of polyunsaturated fatty acids is not random, since it is under genetic control. Moreover, the position of fatty acyl moiety affects their bioavailability in animals and humans.^55^ In this part of our study, we aimed to synthesize and analyze the regiostructured USU type TAG possessing VLC-PUFAs in *sn*-1 and *sn*-3 positions along with octanoic (C8:0)/decanoic (C10:0) acids occupying the *sn*-2 position. Reversely structured SUS type TAG with VLC-PUFAs located in the *sn*-2 position was examined as well.

For our purposes, commercial immobilized nonspecific (Novozyme 435), 1,3-regiospecific (Lipozyme RM IM, Lipozyme TL IM, lipase B *Candida antarctica*, expressed in *Aspergillus oryzae*), and 2-regiospecific (lipase A *Candida antarctica*, expressed in *Aspergillus oryzae*) lipases were employed as biocatalysts to perform interesterification reactions of 3.43 equiv (mole) of ethyl *all-cis*-octacosa-10,13,16,19,22,25-hexaenoate (C28:6 *n*-3, 98%) with medium-chain TAG (MCT, 1.0 equiv) under nonaqueous conditions at 40 °C for 40 h. It can be assumed that the reaction time was long enough to reach thermodynamic equilibrium, and thus, the fatty acyl composition is representative. The MCT reactant (99%), which was separated from free fatty acid residues by column chromatography prior to interesterification, was composed of octanoic (C8:0) and decanoic (C10:0) acids in a ratio of 72:28 (w/w). Major TAG 8–8–10 accounted for 45%, while TAG species 8–8–8 and 8–10–10 constituted 39% and 16%, respectively, as determined by HT-GC/FID. Purified products of enzymatic interesterifications were quantified by high resolution APCI-MS and ^1^H NMR spectroscopy techniques after the addition of ethyl hexacosanoate (99%) and synthetic 1,2-dilauroyl-3-myristoyl-*rac*-glycerol (12–12–14; 99%) as internal standards. Commercial nonspecific and 1,3-regiospecific lipases successfully incorporated C28:6 *n*-3 acyl moieties into the outer positions of the MCT glycerol backbone, but we observed significant differences in the extent of C8:0 and C10:0 substitutions.

**Figure 6:**
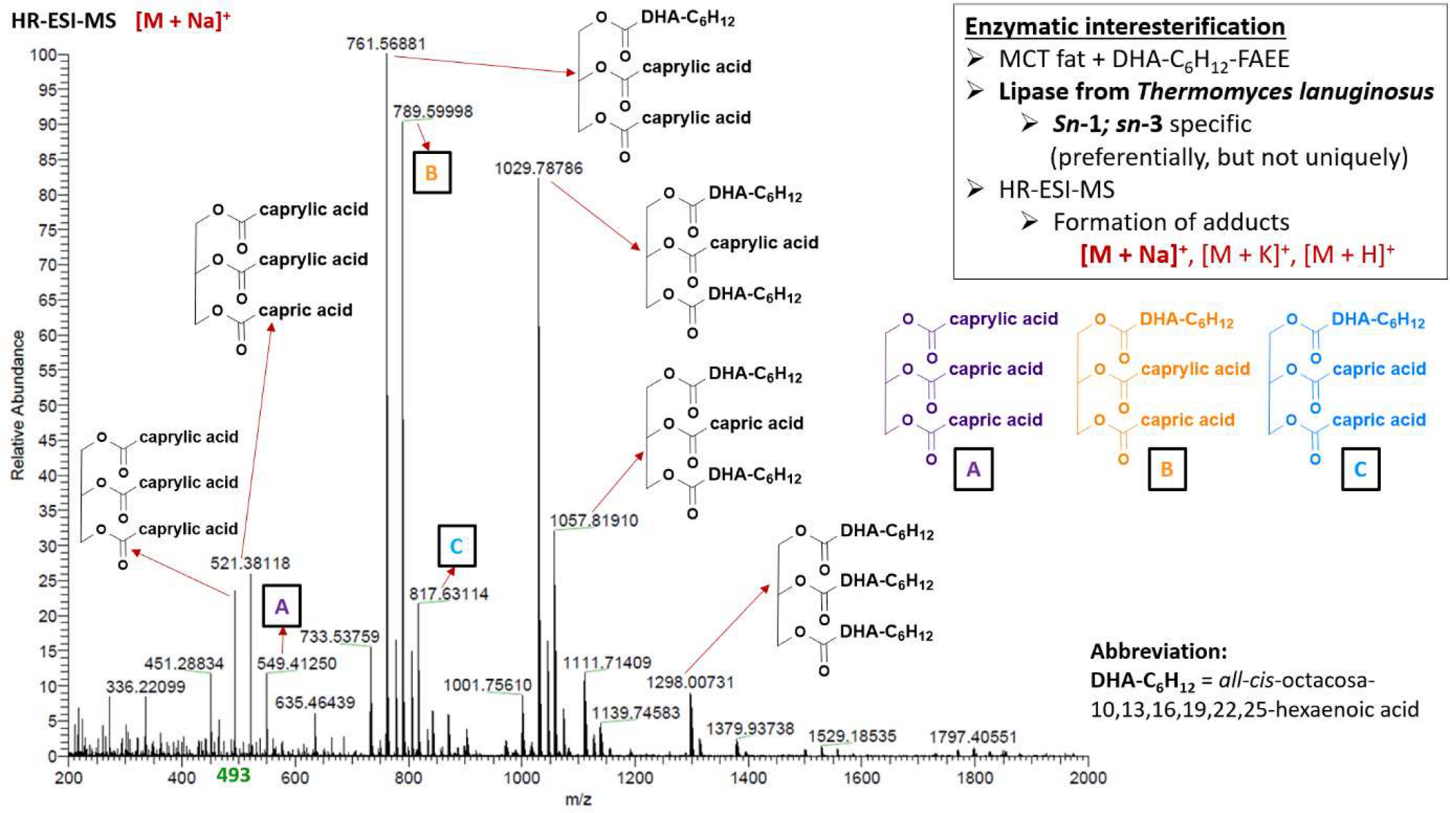
High resolution ESI-MS spectrum of mono-, di-, and tripolyunsaturated TAG species [M + Na]^+^ enriched with *all-cis*-octacosa-10,13,16,19,22,25-hexaenoic acid after 1,3-regiospecific enzymatic interesterification using Lipozyme TL IM.

In the typical example of high resolution ESI-MS spectra (Figure 6) obtained after enzymatic interesterification by Lipozyme TL IM, we observed a substantial amount of monopolyunsaturated TAGs accompanied by less frequent dipolyunsaturated species and minor 1,2,3-tri-(*all-cis*-octacosa-10,13,16,19,22,25-hexaenoyl)glycerol (28:6–28:6–28:6). Among the monounsaturated (USS type) triacylglycerols, the following compounds were identified: 1-*all-cis*-octacosa-10,13,16,19,22,25-hexaenoyl-2,3-dicapryloylglycerol (28:6–8–8), 1-*all-cis*-octacosa-10,13,16,19,22,25-hexaenoyl-2-capryloyl-3-caprinoylglycerol (28:6–8–10), 1-*all-cis*-octacosa-10,13,16,19,22,25-hexaenoyl-2-caprinoyl-3-capryloylglycerol (28:6–10–8), and 1-*all-cis*-octacosa-10,13,16,19,22,25-hexaenoyl-2,3-dicaprinoylglycerol (28:6–10–10), as well as dipolyunsaturated SUS type species comprised of 1,3-di-*all-cis*-octacosa-10,13,16,19,22,25-hexaenoyl-2-capryloylglycerol (28:6–8–28:6), and 1,3-di-*all-cis*-octacosa-10,13,16,19,22,25-hexaenoyl-2-caprinoylglycerol (28:6–10–28:6).

**Figure 7:**
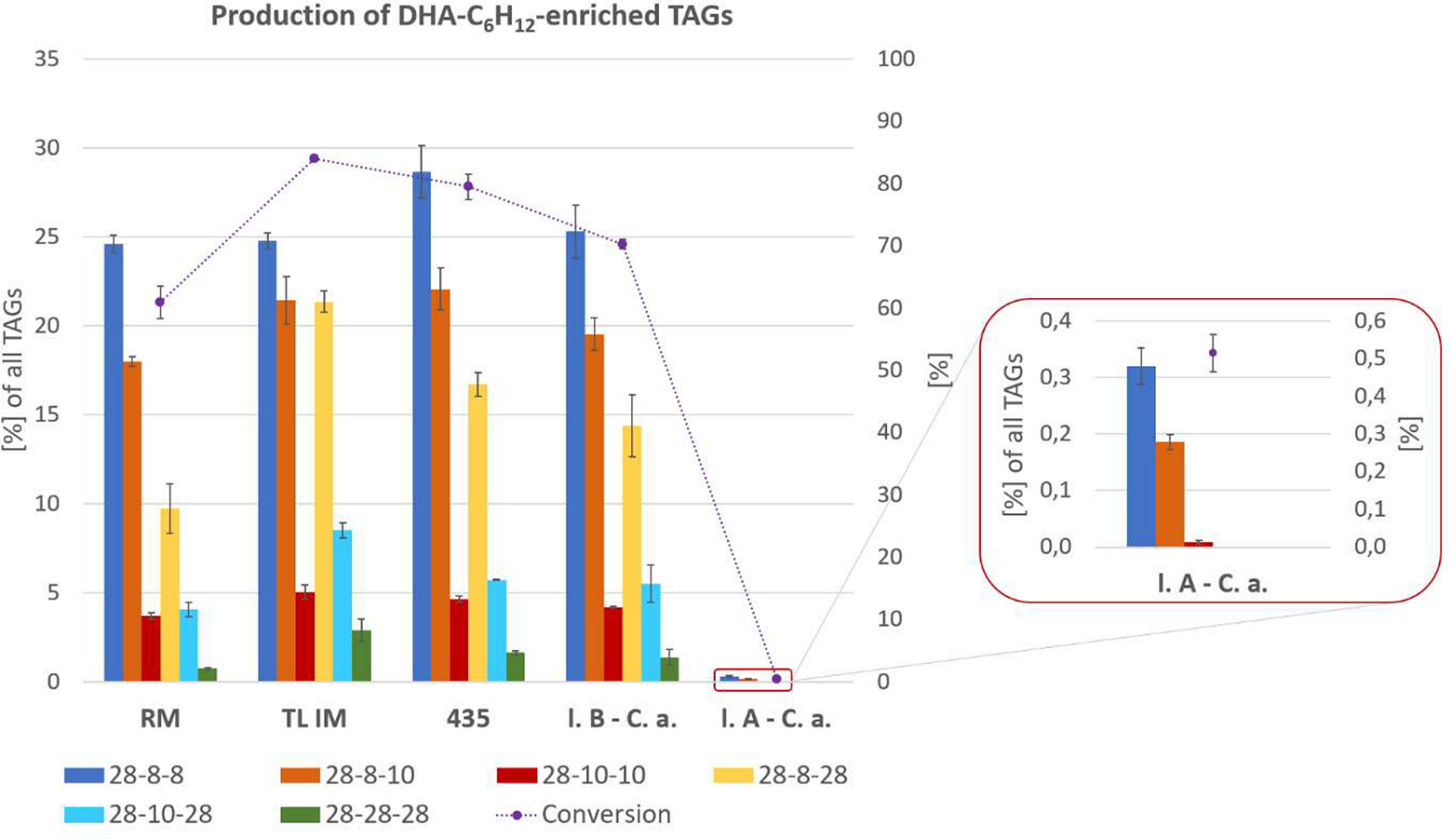
Conversion of commercial medium-chain triacylglycerols during enzymatic interesterification with ethyl *all-cis*-octacosa-10,13,16,19,22,25-hexaenoate (C28:6 *n*-3) using 1,3-regiospecific, nonspecific, and 2-regiospecific lipases.

We demonstrated the efficient conversion of MCT offered by the Lipozyme TL IM, reaching up to 84% yield. Major USS type triacylglycerols with prevailing 28–8–8 (25%), which could be formed from both dominant precursors 8–8–10 and 8–8–8, accounted for 51%. The *Thermomyces lanuginosus* lipase also provided the highest production of dipolyunsaturated 28:6–8–28:6 (21%) and 28:6–10–28:6 (9%) triacylglycerols as well as the fully substituted 28:6–28:6–28:6 (3%) reaction product. In fact, TL IM lipase was characterized by the highest water content (4.69%), since it was immobilized on a hygroscopic SiO_2_ carrier, as confirmed by the results of FTIR analysis. The FTIR spectrum (Figure S9) displayed strong absorption bands of water and SiO_2_ with peaks at 3319 cm^–1^, 1648 cm^–1^, 1073 cm^–1^, and 798 cm^–1^ assigned to OH vibrations in water and Si–O–Si asymmetric and symmetric stretch vibrations, respectively. The combined effect of excessive moisture content along with the acidic support of the enzyme with a high specific surface area resulted in considerable interesterification of the terminal positions but also greatly promoted undesired acyl migration to the *sn*-2 position. When the *Thermomyces lanuginosus* lipase was replaced by nonspecific Novozyme 435 (Figure S10) and 1,3-regiospecific lipase B from *Candida antarctica* (Figure S11) or Lipozyme RM IM (Figure S12) with a decreasing moisture content (Table 1) under the same experimental setup, the conversion of medium chain TAG shown in Figure 7 decreased to 80%, 70%, and 61%, respectively. At the same time, migration of the acyl group was also suppressed (1–2%). There was a demonstrable relationship between the moisture content of the commercial enzyme (0.70–4.69%) and the achieved conversion, including acyl migration. From a practical point of view, the selection of a suitable lipase is a necessary compromise between the overall conversion of MCT sample and the yield of individual reaction products, especially USU (46–55%) and USS (10–21%) type TAG. In the coming months, we will focus on the determination of reaction kinetics as another criterion for enzyme selection.

**Figure 8:**
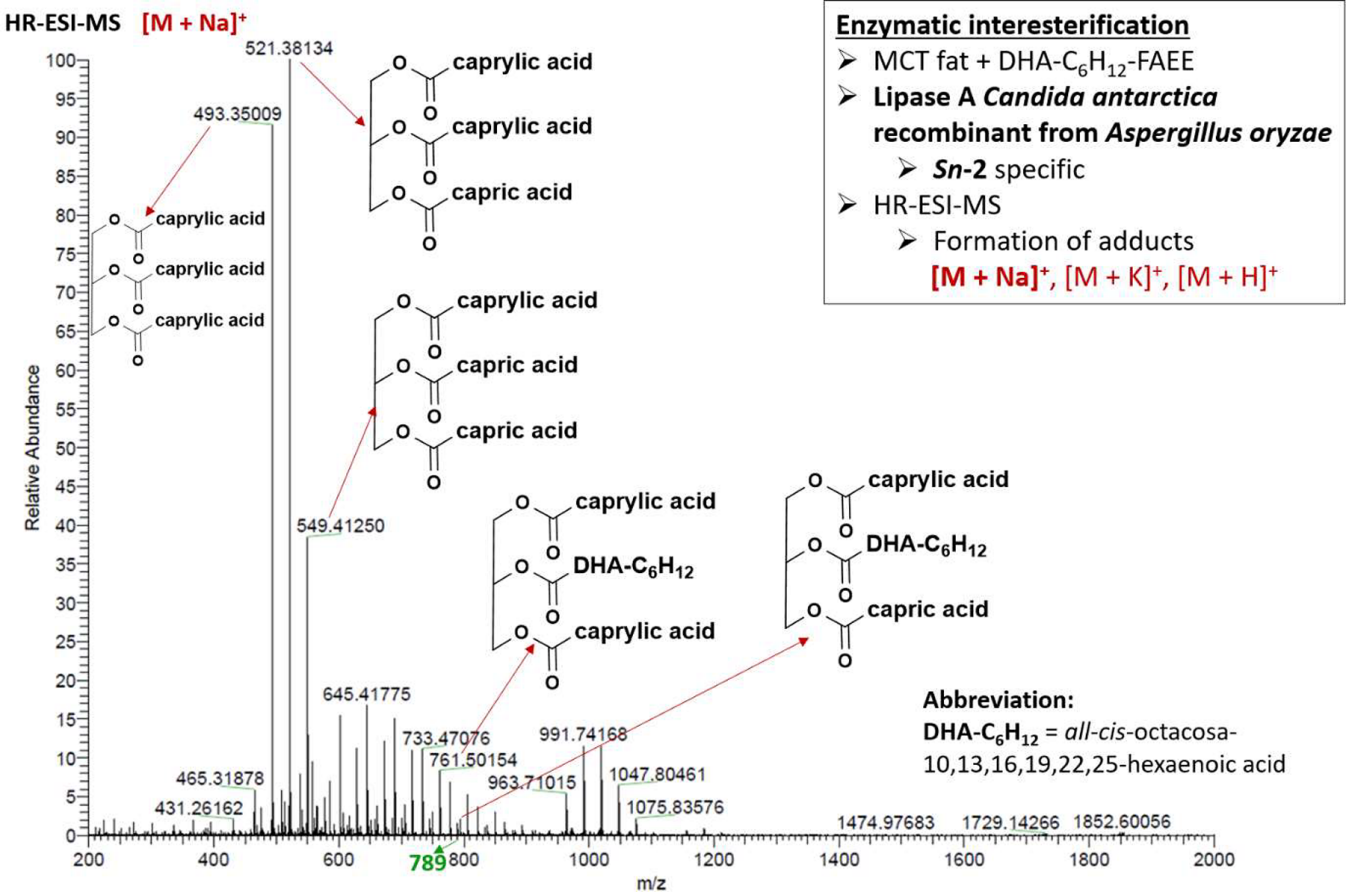
High resolution ESI-MS spectrum of monopolyunsaturated TAG species [M + Na]^+^ enriched with *all-cis*-octacosa-10,13,16,19,22,25-hexaenoic acid after 2-regiospecific enzymatic interesterification using lipase B *Candida antarctica*.

Once we developed the 1,3-regioselective enzymatic protocol that allowed us to insert unique VLC-PUFAs for the first time into structured TAGs (USU type), we tried to prepare reversely structured TAG (SUS type) with C28:6 *n*-3 exclusively in the *sn*-2 position as shown in Figure 8. A mass spectra obtained by ESI-MS allowed us to determine the accurate mass of Na^+^ adduct ions [M + Na]^+^ of 1,3-dicapryloyl-2-*all-cis*-octacosa-10,13,16,19,22,25-hexaenoylglycerol (8–28:6–8) and 1,3-dicaprinoyl-2-*all-cis*-octacosa-10,13,16,19,22,25-hexaenoylglycerol (10–28:6–10) at *m*/*z* 761.50154 and 789.53230, respectively. It should, however, be noted that 2-regiospecific lipase B from *Candida antarctica* displayed a much lower tolerance to polyunsaturated *all-cis*-octacosa-10,13,16,19,22,25-hexaenoate than other lipases.

### 3.4 New Insights into Enzymatic Interesterification of VLC-PUFAs with Glycerophosphorylcholine

Organic synthesis of structured phosphatidylcholine cannot proceed without the multistep introduction of protective groups followed by their cleavage, so the direct insertion of unstable VLC-PUFAs by commercial lipases represents a faster and greener alternative. The question is whether the applied enzymes are versatile enough to tolerate both substrates during interesterification experiments as described in the literature.^56^ Data on the kinetics and selectivity of designed enzymatic interesterification are unknown. For our purposes, the most successful 1,3-regiospecific (Lipozyme RM IM, Lipozyme TL IM) lipases were employed as biocatalysts to perform nonaqueous interesterification reactions of 5.5 equiv (mole) of ethyl *all-cis*-hexacosa-11,14,17,20,23-pentaenoate (C26:5 *n*-3, 98%) with 1,2-dipalmitoyl-*sn*-glycero-3-phosphorylcholine (DPPC, 1.0 equiv), simulating commercially available lecithin. The reactions proceeded at 45 °C for 48 h under inert conditions. Purified products of enzymatic interesterifications were qualitatively characterized by high resolution ESI-MS. Moreover, equilibrium composition of fatty acids present in glycerophospholipids was quantitatively determined by GC-FID analysis after their SPE cleanup using our developed elution system.

**Figure 9:**
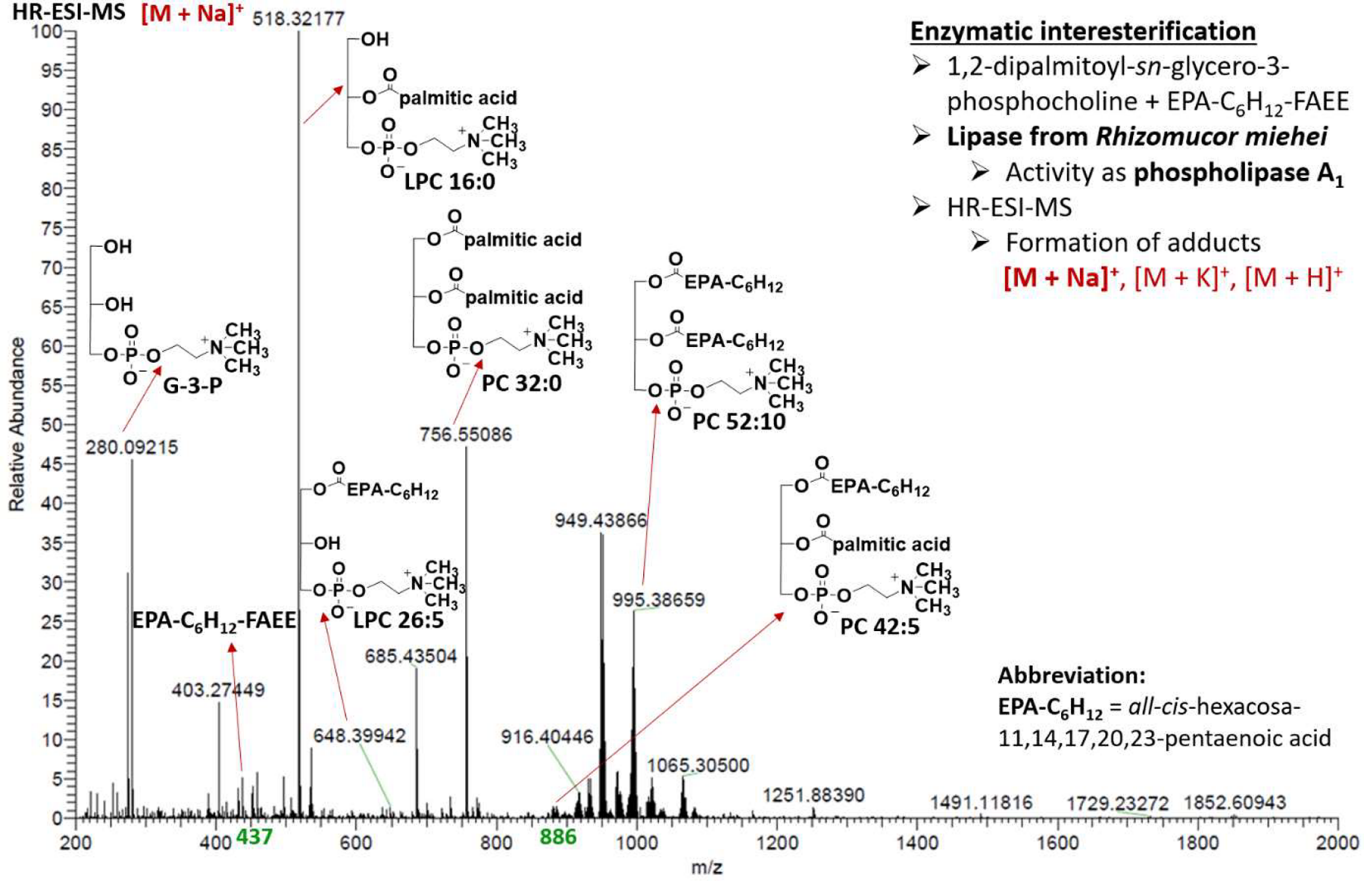
High resolution ESI-MS spectrum of mono- and dipolyunsaturated glycerophoshatidylcholine species [dominant M + Na]^+^ enriched with *all-cis*-hexacosa-11,14,17,20,23-pentaenoic acid after regiospecific enzymatic interesterification using Lipozyme RM IM.

Our attempt to prepare phosphatidylcholine enriched with C26:5 *n*-3 using Lipozyme TL IM with the highest moisture content (4.69%) resulted in low conversion due to preferential hydrolysis of DPPC. The results clearly demonstrated that surface-active glycerophospholipids with a strong affinity to polar silica gel undergo mainly competitive hydrolysis in the presence of excessive H_2_O. On the other hand, when the *Thermomyces lanuginosus* lipase was replaced by immobilized *Rhizomucor miehei* (Lipozyme RM IM) lipase with a moisture content of 0.70% under the same experimental conditions, the conversion of glycerophospholipids significantly increased as shown in Figure 9. Our results of FTIR analysis confirmed the presence of different immobilization carriers, namely, macroporous ion-exchange resin (Figure S13). The FT-IR spectrum displayed strong absorption bands at 1726 and 2949 cm^–1^ assigned to the C=O stretching vibration of the saturated ester group and aliphatic C–H stretching, respectively. The strong peak at 1141 cm^–1^ corresponded to the v_s_ (COC) of the ester linkage.

Further characterization of Lipozyme RM IM reaction products performed by HR-ESI-MS confirmed the formation of 1-*all-cis*-hexacosa-11,14,17,20,23-pentaenoyl-2-palmitoyl-*sn*-glycero-3-phosphorylcholine (PC 42:5) and 1,2-di-*all-cis*-hexacosa-11,14,17,20,23-pentaenoyl-*sn*-glycero-3-phosphorylcholine (PC 52:10) accompanied by hydrolysis side products such as 1-*all-cis*-hexacosa-11,14,17,20,23-pentaenoyl-*sn*-glycero-3-phosphorylcholine (2-LPC 26:5), 2-palmitoyl-*sn*-glycero-3-phosphorylcholine (1-LPC 16:0), and glycerophosphorylcholine (G-3-P). The involvement of the *sn*-2 position in the preparation of structured phosphatidylcholine is advantageous compared to structured TAGs and can be used effectively to maximize the substitution of bioactive VLC-PUFAs for palmitic acid from DPPC in a single step. Intramolecular fatty acyl-migration was most likely promoted by the long reaction time (48 h), high interesterification temperature (45 °C), and the presence of protons from the ion-exchange resin of the *Rhizomucor miehei* lipase. First of all, 1,3-regiospecific lipase (Lipozyme RM IM) attacked the *sn*-1 position of DPPC yielding lysophosphatidylcholine (1-LPC 16:0) and interesterification products (PC 42:5) simultaneously. Over time, a thermodynamic equilibrium was established that involved the migration of acyls to the *sn*-2 position. The formation of 1-palmitoyl-2-*all-cis*-hexacosa-11,14,17,20,23-pentaenoyl-*sn*-glycero-3-phosphorylcholine (PC 42:5) proceeded via cyclic five-membered hemiortho ester type intermediate as previously described.^57^ Finally, the desired dipolyunsaturated species (PC 52:10) could be reached either by prominent interesterification of PC 42:5 or less likely by reesterification of 1-LPC 26:5. When using 5.5 equiv of ethyl *all-cis*-hexacosa-11,14,17,20,23-pentaenoate, calculated equilibrium composition of VLC-PUFAs present in phosphatidylcholine is 80.6% when both *sn*-1 and *sn*-2 positions participate in interesterification. The composition of the fatty acids differed slightly, with VLC-PUFAs represented only by 63.6%; the deviation was probably caused by the presence of interfering lysophosphatidylcholine with the palmitoyl moiety in the *sn*-1 or *sn*-2 positions. To conclude, we have successfully prepared all bioavailable forms of bound VLC-PUFAs including their ethyl esters, triacylglycerols, and phosphatidylcholines using the more sustainable chemo-enzymatic method from readily available starting materials.

VLC-PUFA enriched ethyl esters, triacylglycerols, or phosphatidylcholines can be used in clinical trials to test their efficacy for the treatment of blinding eye diseases and male infertility. On the surface, one may argue that since the enzyme ELOVL4, which makes VLC-PUFAs, exists in tissues that need VLC-PUFAs, why not use dietary supplementation seafood or fish oil-containing supplements that can then be elongated to VLC-PUFAs in the body by the ELOVL4 enzyme. While this reasoning may apply to people without ELOVL4 mutations, it will not apply to STGD3 patients who have defective ELOVL4 enzymatic function. In addition, it must also be noted that as we age epigenetic modification reduces the ability of the fatty acid elongases that elongate C20–C22 PUFAs to generate precursors for ELOVL4, which may explain decreased retinal levels of VLC-PUFAs as we age, independent of ELOVL4 mutations. Therefore, there is a need to explore alternative avenues for VLC-PUFAs supplementation or therapy. Given the morbidities of Stargardt-like macular dystrophy and age-related macular degeneration, the development of an oral product that could provide the missing or reduced VLC-PUFAs to the retina would be a tremendous step forward in the treatment of these two devastating diseases. Likewise, VLC-PUFAs may prove to be a simple and effective means to treat some forms of male infertility.

## Supporting Information Available

Figures S1 and S2, structural analyses of **1a** and **2a**; Figure S3, overall process; Figures S4 and S5, reaction schemes and calculations; Figure S6, HR-APCI-MS and EI-MS analyses of **3b**; Figure S7, ^1^H NMR analysis of **3b**; Figure S8, mass spectra of minor isomers of ethyl hexacosapentaenoate; Figure S9, FT-IR spectra of immobilized *Thermomyces lanuginosus* lipase; Figures S10–S12, high resolution ESI-MS spectrum of mono-, di-, and tripolyunsaturated TAG 44 species; Figure S13, FTIR spectra of immobilized *Rhizomucor miehei* (PDF)

## Abbreviations Used

LC-PUFAs: long-chain polyunsaturated fatty acids
AA: arachidonic acid
EPA: eicosapentaenoic acid
DHA: docosahexaenoic acid
PUFAs: polyunsaturated fatty acids
VLC-PUFAs: very-long-chain polyunsaturated fatty acids
VLC-FAs: very-long-chain fatty acids
ELOVL4: very-long-chain fatty acids-4 enzyme
STGD3: Stargardt macular dystrophy
AMD: age-related macular degeneration
PL: glycerophospholipid
TAG: triacylglycerol
TLC: thin-layer chromatography
EI-MS: electron impact mass spectrometry
FAME: fatty acid methyl ester
FAEE: fatty acid ethyl ester
DHA FAEE: *all*-*cis*-docosa-4,7,10,13,16,19-hexaenoic acid ethyl ester
EPA FAEE: *all*-*cis*-eicosa-5,8,11,14,17-pentaenoic acid ethyl ester
GC-FID: gas chromatography coupled with flame-ionization detector
GC-MS: gas chromatography coupled with mass spectrometry
ESI: electrospray ionization
APCI: atmospheric pressure chemical ionization
