# Supplementary material for: Novel Approaches for Elongation of Fish Oils into Very-Long-Chain Polyunsaturated Fatty Acids and Their Enzymatic Interesterification into Glycerolipids
Source: J Agric Food Chem. 2023 Nov 10;71(46):17909–23. doi: 10.1021/acs.jafc.3c05355 (PMC10682991; doi:10.1021/acs.jafc.3c05355)
Supplement: Supplementary file 1 — jf3c05355_si_001.pdf [file jf3c05355_si_001.pdf]

TITLE AND AUTHORSHIP

Title:

Novel Approaches for Elongation of Fish Oils into Very-Long-Chain Polyunsaturated Fatty Acids and Their Enzymatic Interesterification into Glycerolipids

Authors:

Tereza Honzíková[1], Martin-Paul Agbaga[2,3], Robert Eugene Anderson[2,3], Richard Brush[2], Mohiuddin Ahmad[2], Lenka Musílková[4], Karolína Šejstalová[4], Katsiaryna Alishevich[1], Radek Beneš[1], Petra Šimicová[1], Markéta Berčíková[1], Vladimír Filip[1], Jan Kyselka[1]

Affiliations and addresses of the authors:

[1]Department of Dairy, Fat and Cosmetics, Faculty of Food and Biochemical Technology, University of Chemistry and Technology, Technická 3, 166 28 Prague, Czechia

[2]Departments Of Cell Biology & [3]Ophthalmology, Dean McGee Eye Institute, University of Oklahoma Health Sciences Center, Oklahoma City, OK 73104, USA

[4]The Department of Chemistry of Natural Compounds, Faculty of Food and Biochemical Technology, University of Chemistry and Technology, Technická 5, 166 28 Prague, Czechia

Email address of the corresponding authors: kyselkaj@vscht.cz

Tel: 220 443 266

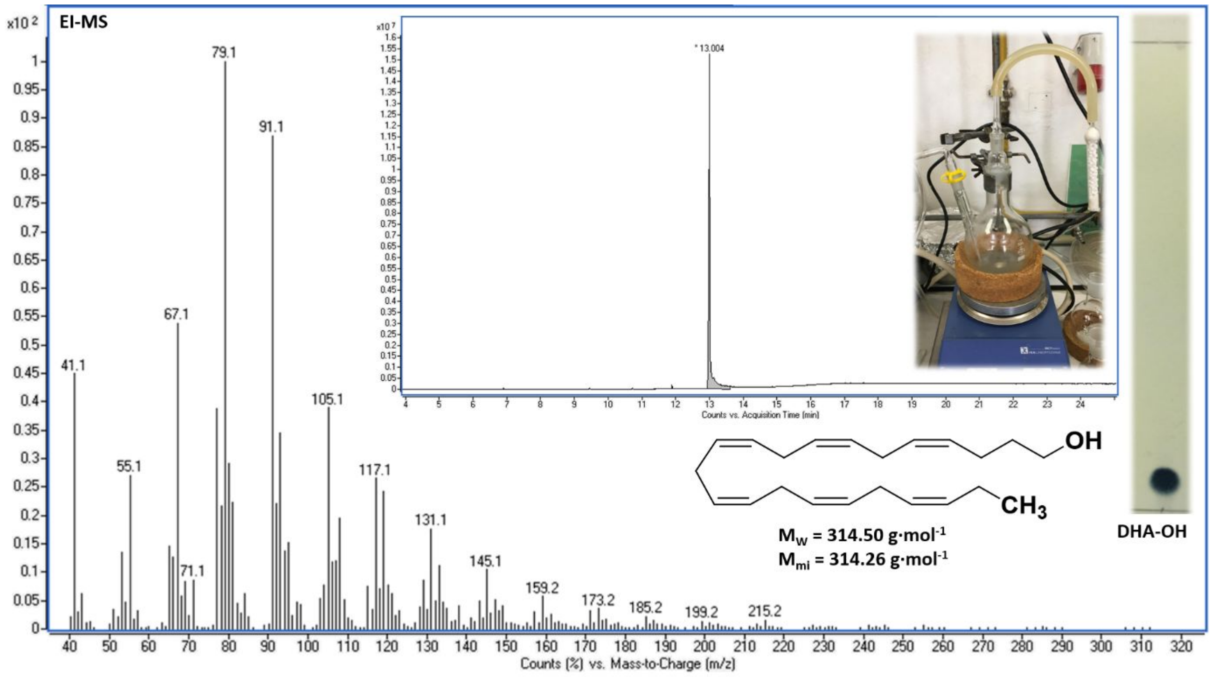

Figure S1. Mass spectra (EI-MS), GC-based separation of *all*-*cis*-docosa-4,7,10,13,16,19-hexaen-1-ol (**1a**) on a 5% phenylmethyl polysiloxane column (upper image), thin-layer chromatography results (left image) developed in a solvent system of hexanes:Et_2O:formic acid (80:20:2; v/v/v) with universal phosphomolybdic acid detection.

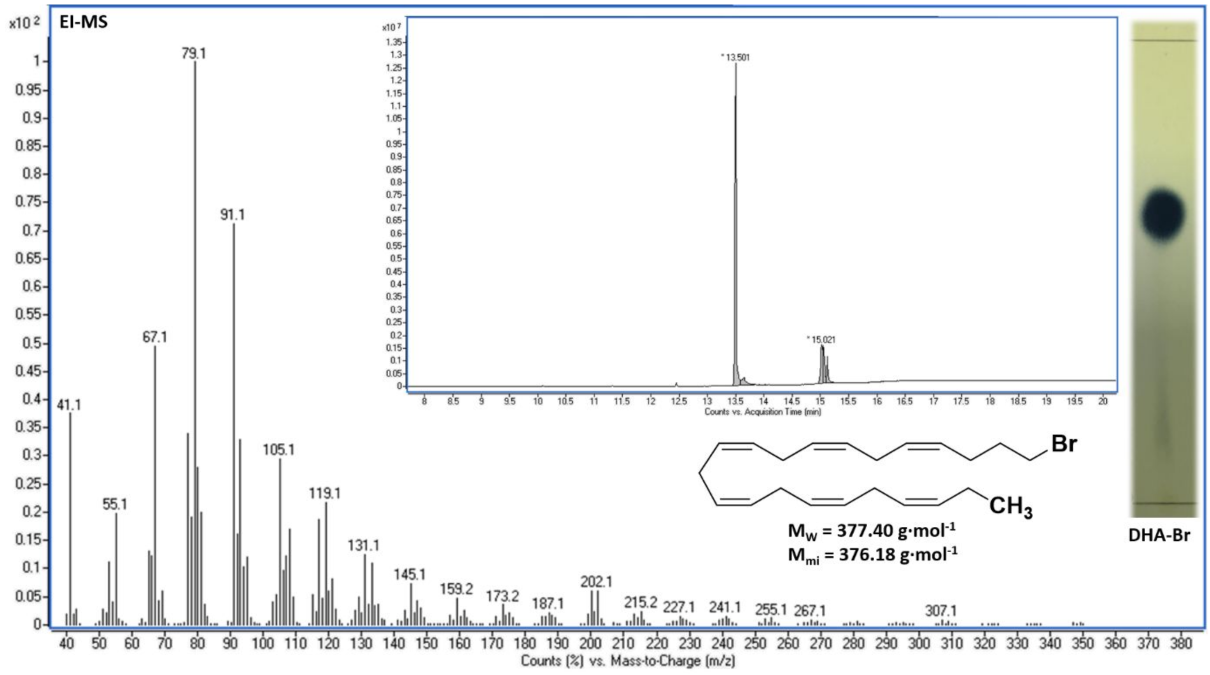

Figure S2. Mass spectra (EI-MS), GC-based separation of *all*-*cis*-docosa-4,7,10,13,16,19-hexaenyl bromide (**2a**) on a 5% phenylmethyl polysiloxane column (upper image), thin-layer chromatography results (left image) developed in a solvent system of hexanes:Et_2O:formic acid (80:20:2; v/v/v) with universal phosphomolybdic acid detection.

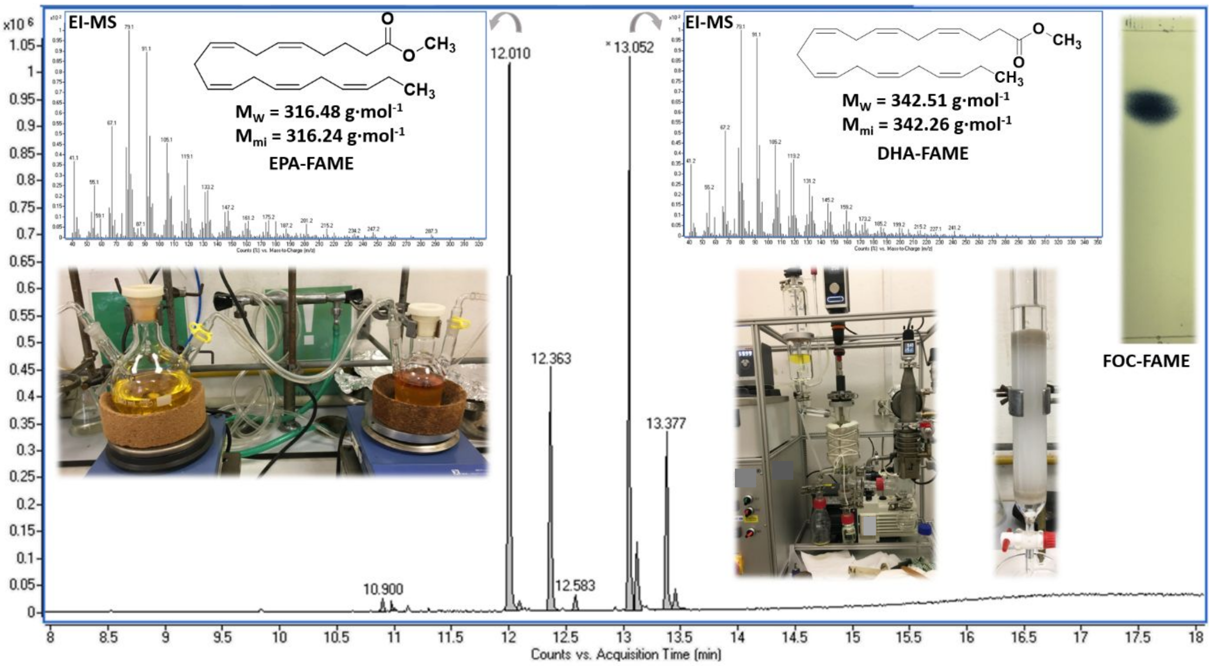

Figure S3. Mass spectra (EI-MS), GC-based separation of fish oil fatty acid methyl esters on a 5% phenylmethyl polysiloxane column (lower image), thin-layer chromatography results (left image) developed in a solvent system of hexanes:Et_2O:formic acid (80:20:2; v/v/v) with universal phosphomolybdic acid detection.

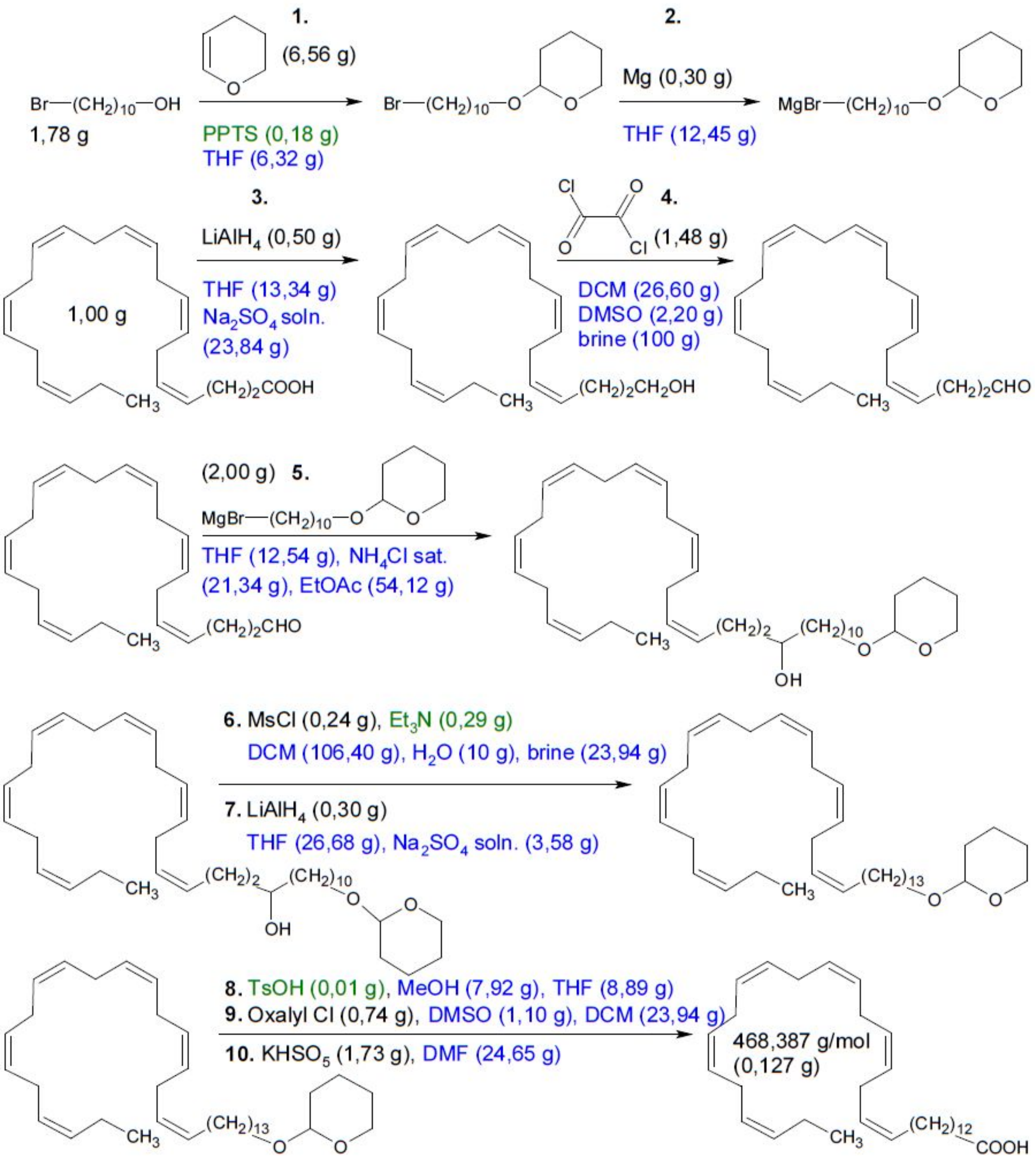

Atom Economy (%) = $100 \times M_{products}/\Sigma M_{reactants} = 100 \times (468.387/942.085) =$ **49.72 %**

Process Atom Economy (%) = $100 \times m_{products}/\Sigma m_{reactants} = 100 \times (0.127/14.63) =$ **0.87 %**

$$\textbf{Environmental Factor (g/1 g of product)} = \frac{\Sigma m_{reactants} + \Sigma m_{catalysts} + \Sigma m_{solvents} + \Sigma m_{byproducts}}{m_{product}} =$$

$$= \frac{14.63\text{ g} + 0.48\text{ g} + 509.85\text{ g}}{0.127\text{ g}} = \textbf{4133.54 g/g product}$$

Figure S4. Reaction scheme and green metrics for the synthetic procedure of Goruspundi et al.[32] / Wade et al.[49].

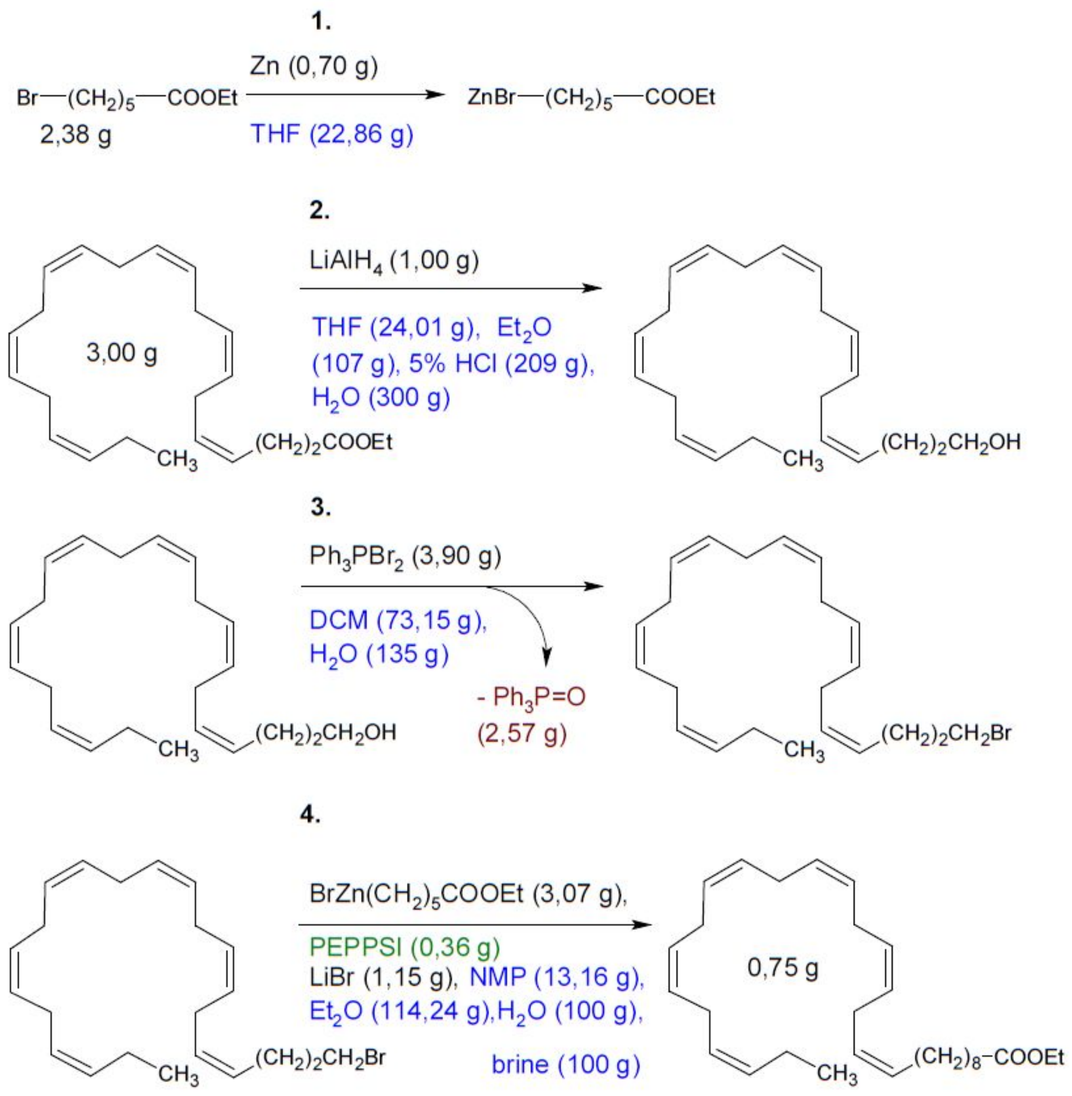

Atom Economy (%) = $100 \times M_{products}/\Sigma M_{reactants} = 100 \times (440.373/835.40) =$ **52.71 %**

Process Atom Economy (%) = $100 \times m_{products}/\Sigma m_{reactants} = 100 \times (0.749/12.134) =$ **6.17 %**

$$\textbf{Environmental Factor (g/1 g of product)} = \frac{\Sigma m_{reactants} + \Sigma m_{catalysts} + \Sigma m_{solvents} + \Sigma m_{byproducts}}{m_{product}} =$$

$$= \frac{12.134\ g + 0.36\ g + 1198.42\ g + 2.57\ g}{0.749\ g} = \textbf{1620.14 g/g}$$

Figure S5. Reaction scheme and green metrics for our developed elongation protocol.

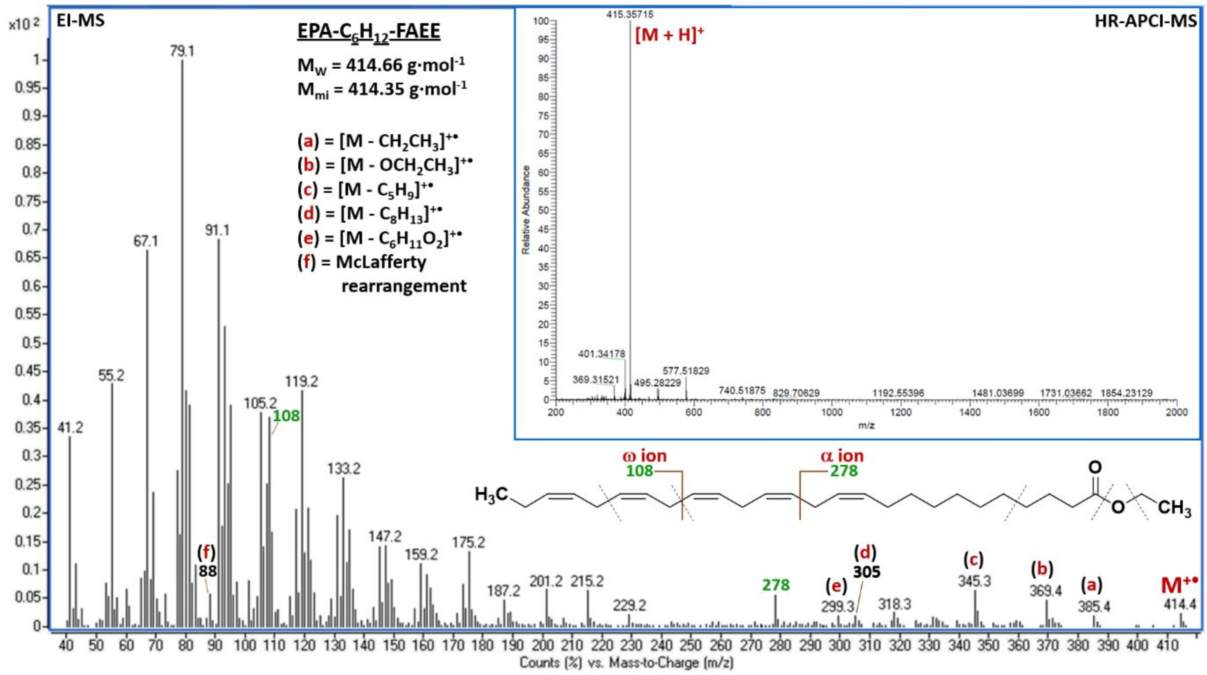

Figure S6. HR-APCI-MS (upper image) and EI-MS (lower image) analysis of ethyl *all-cis*-hexacosa-11,14,17,20,23-pentaenoate (**3b**).

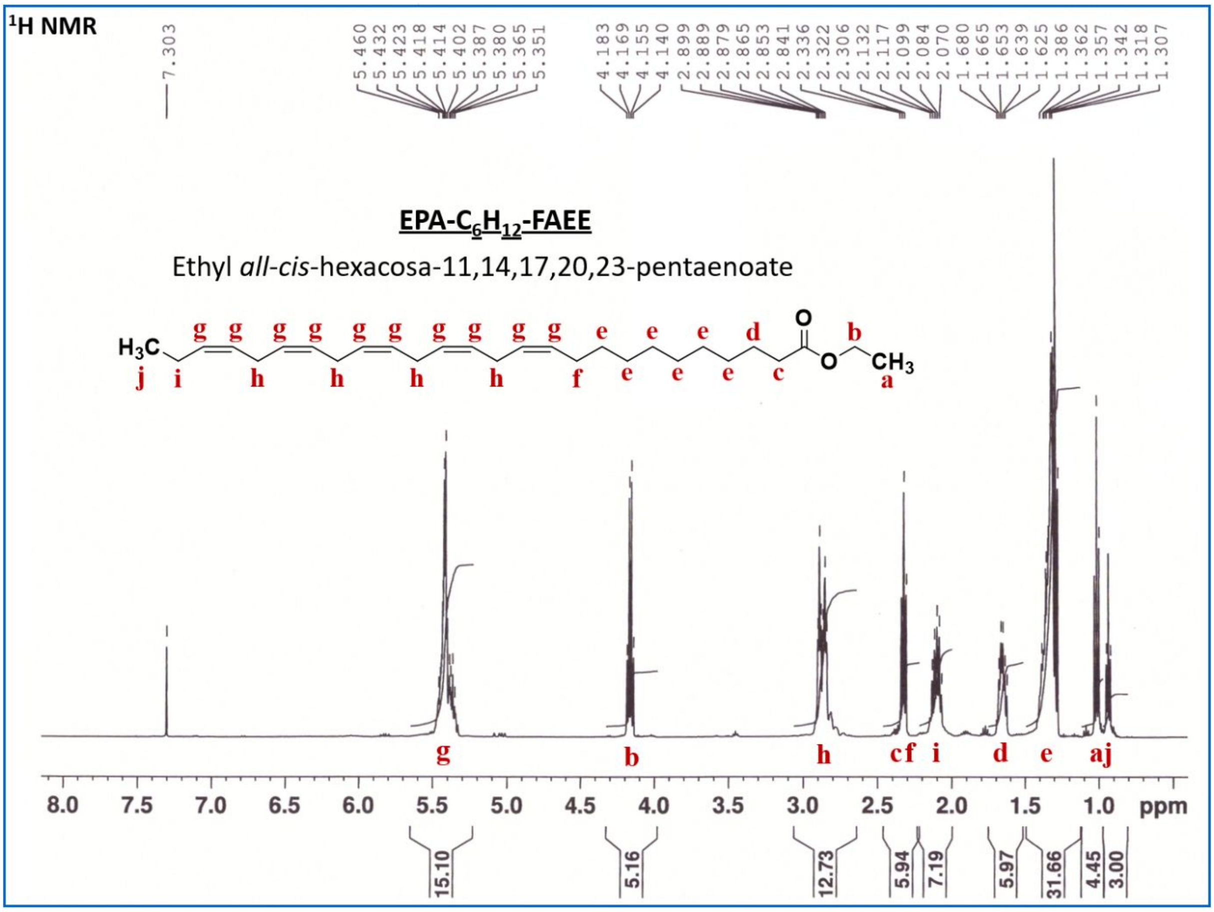

Figure S7. ^{1}H NMR analysis of ethyl *all-cis*-hexacosa-11,14,17,20,23-pentaenoate (**3b**).

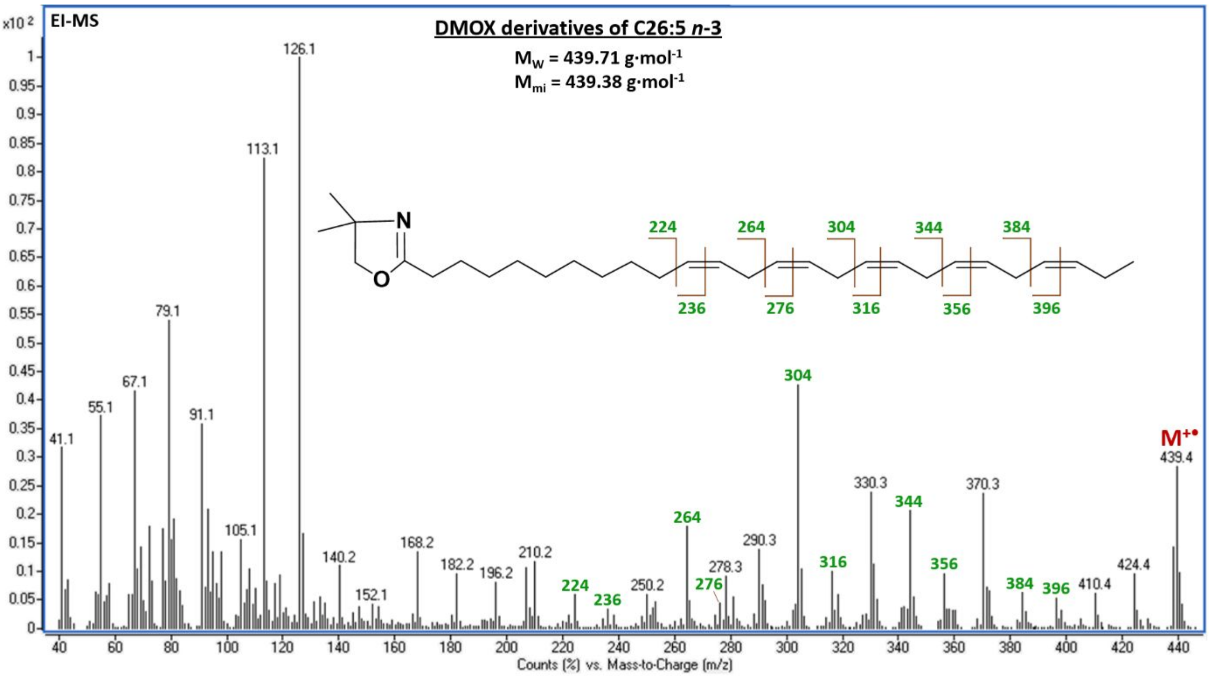

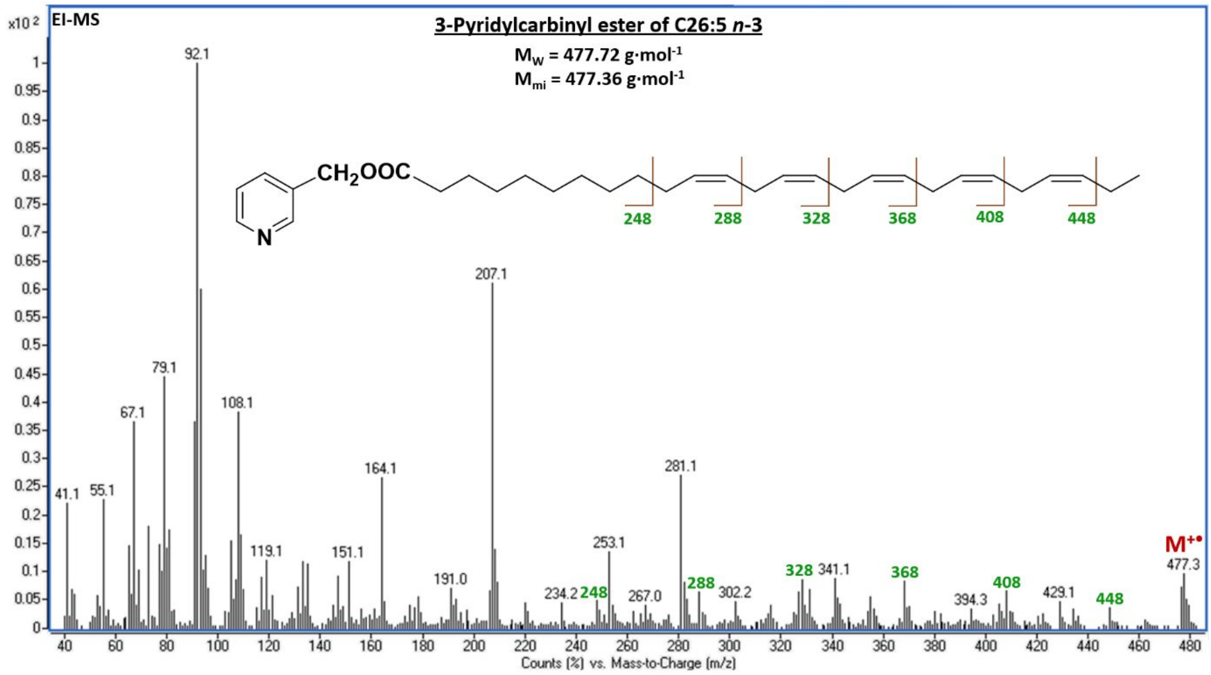

Figure S8. Mass spectra of minor isomers of ethyl hexacosapentaenoate derived from EPA and fish oil concentrates in the form of 4,4-dimethyloxazoline (upper image) and 3-pyridylcarbinol ester (lower image) derivatives.

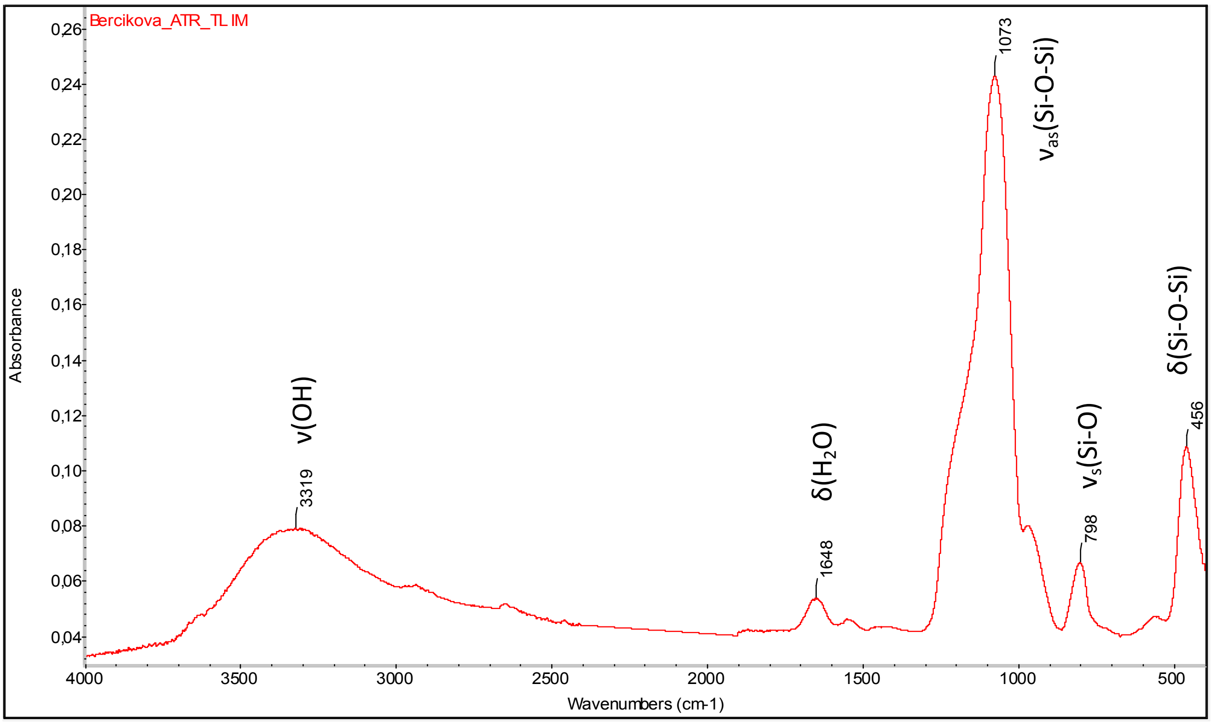

Figure S9. FT-IR spectra of immobilized *Thermomyces lanuginosus* lipase (Lipozyme® TL IM) on SiO_2 carrier.

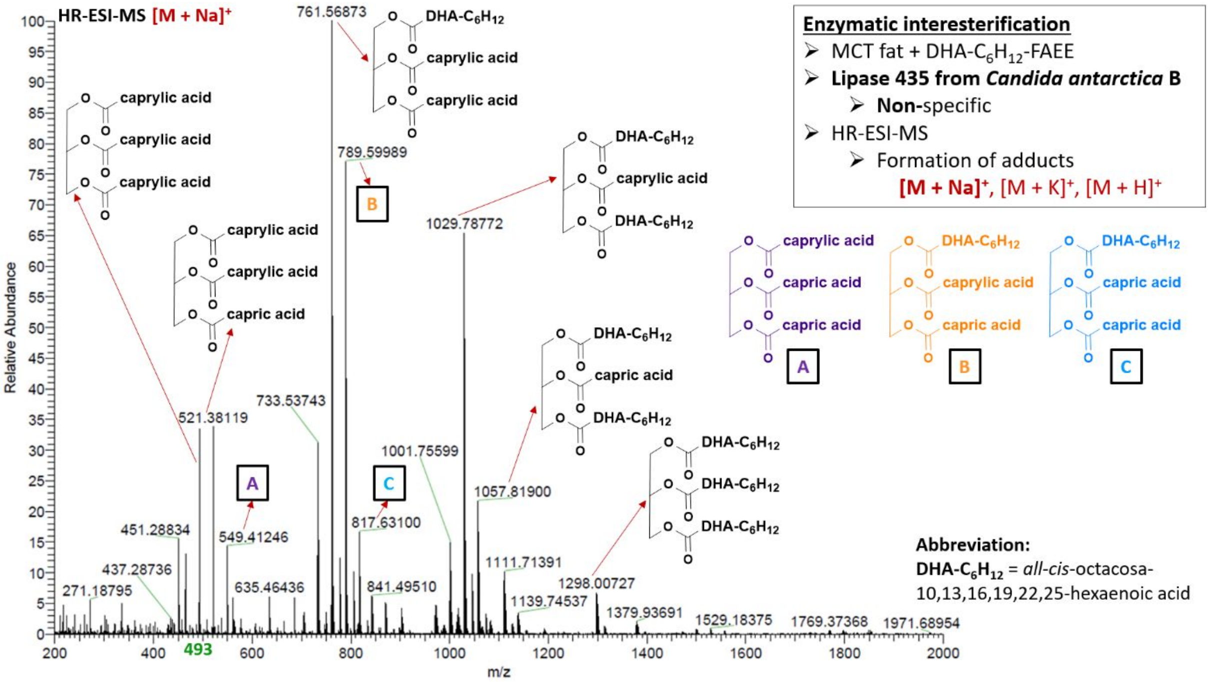

Figure S10. High resolution ESI-MS spectrum of mono-, di-, and tripolyunsaturated TAG species $[M+Na]^+$ enriched with *all-cis*-octacosa-10,13,16,19,22,25-hexaenoic acid after non-specific enzymatic interesterification using Novozyme® 435.

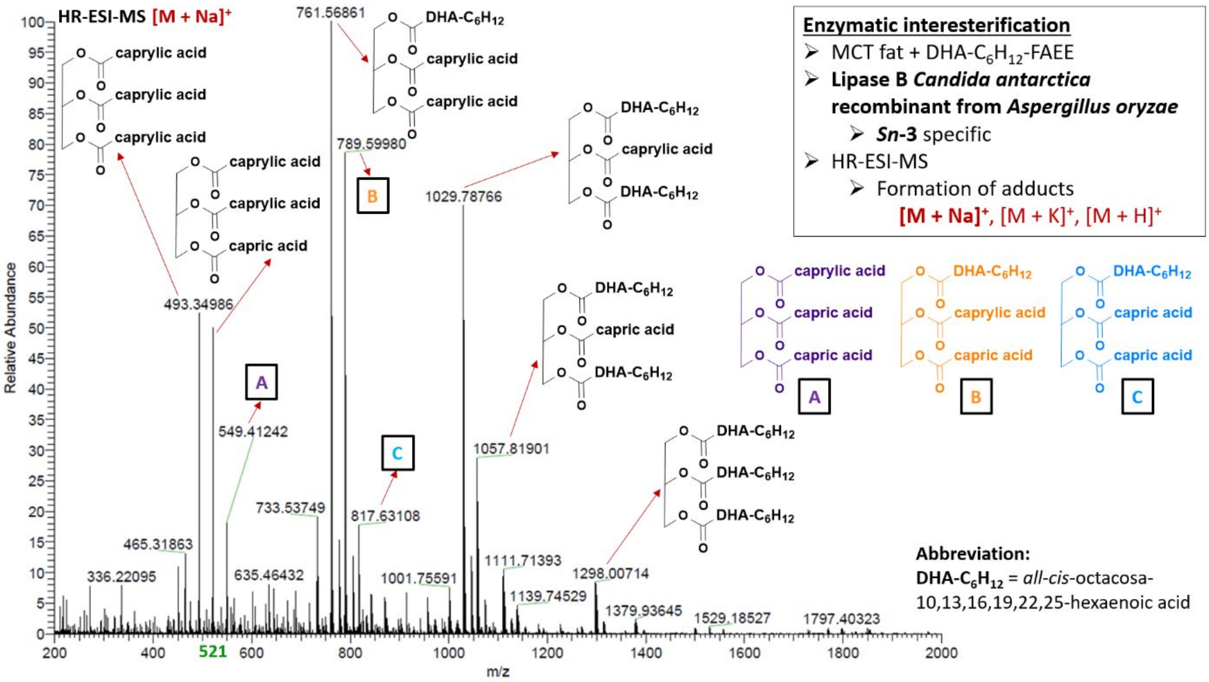

Figure S11. High resolution ESI-MS spectrum of mono-, di-, and tripolyunsaturated TAG species $[M+Na]^+$ enriched with *all-cis*-octacosa-10,13,16,19,22,25-hexaenoic acid after 1,3-regiospecific enzymatic interesterification using lipase B from *Candida antarctica*.

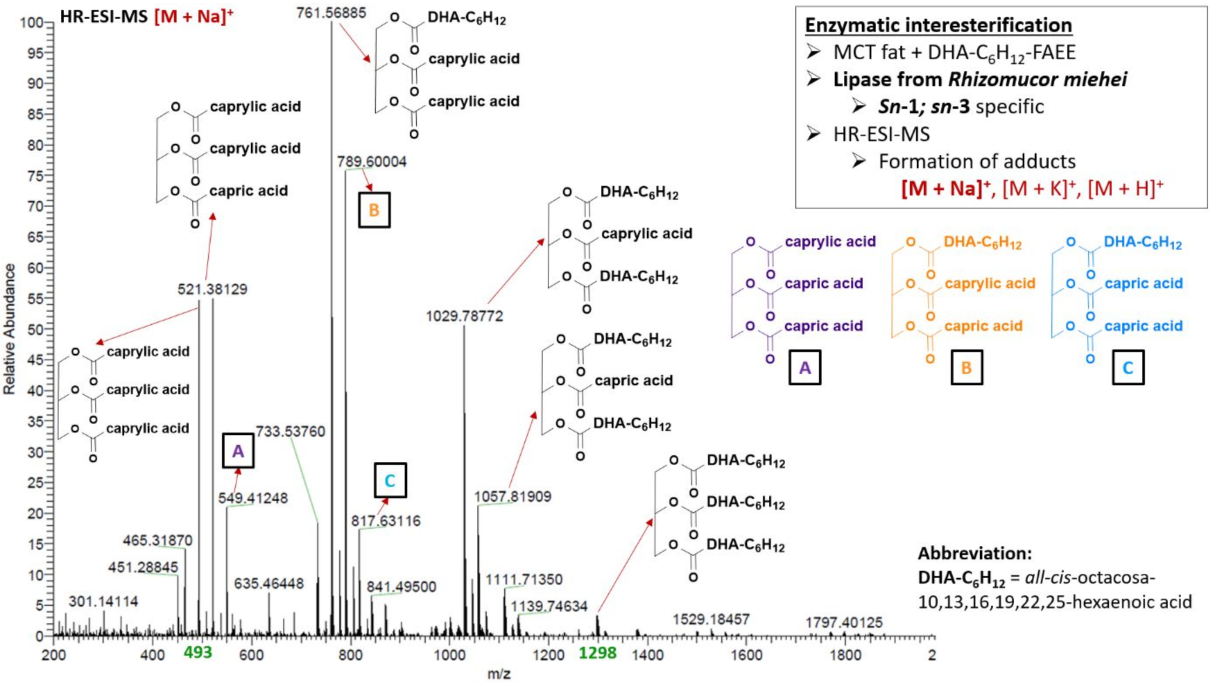

Figure S12. High resolution ESI-MS spectrum of mono-, di-, and tripolyunsaturated TAG species $[M+Na]^+$ enriched with *all-cis*-octacosa-10,13,16,19,22,25-hexaenoic acid after 1,3-regiospecific enzymatic interesterification using Lipozyme® RM IM.

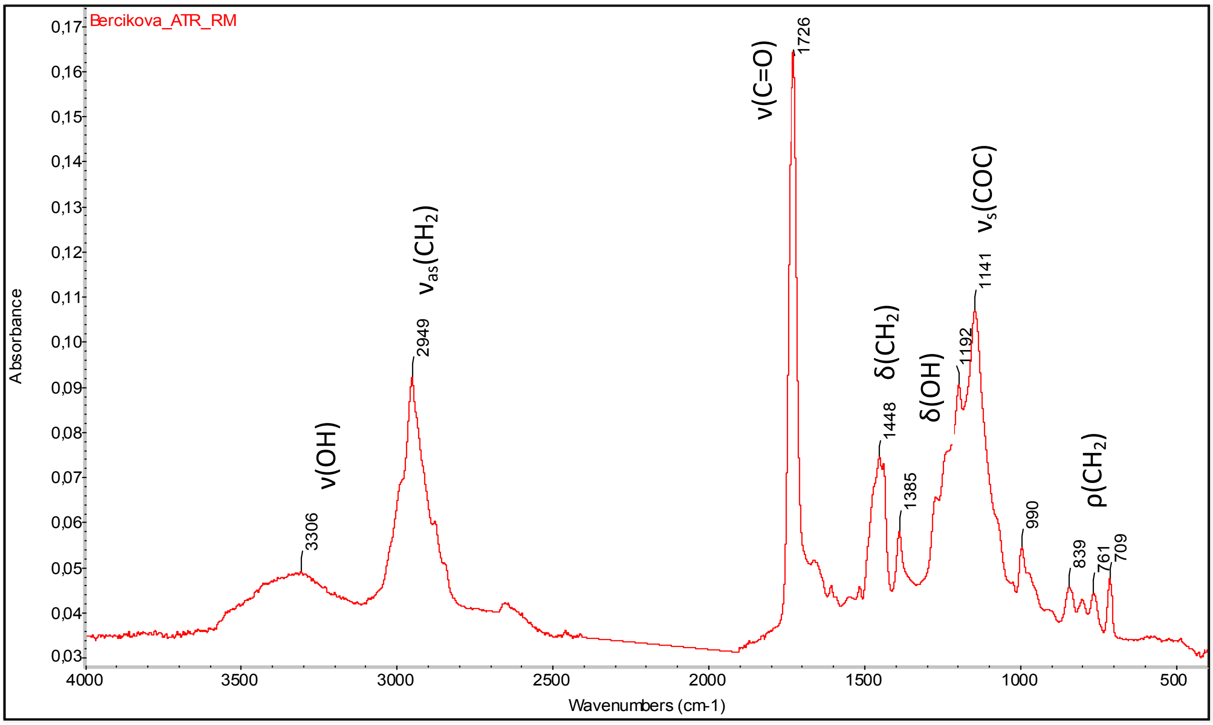

Figure S13. FTIR spectra of immobilized *Rhizomucor miehei* (Lipozyme® RM IM) lipase.
